# Genomic Sequencing of Bacillus cereus Sensu Lato Strains Isolated from Meat and Poultry Products in South Africa Enables Inter- and Intranational Surveillance and Source Tracking

**DOI:** 10.1128/spectrum.00700-22

**Published:** 2022-04-27

**Authors:** Laura M. Carroll, Rian Pierneef, Aletta Mathole, Abimbola Atanda, Itumeleng Matle

**Affiliations:** a Structural and Computational Biology Unit, EMBL, Heidelberg, Germany; b Biotechnology Platform, Agricultural Research Council, Onderstepoort Veterinary Research, Onderstepoort, South Africa; c Bacteriology Division, Agricultural Research Council, Onderstepoort Veterinary Research, Onderstepoort, South Africa; Agriculture and Agriculture-Food Canada

**Keywords:** *Bacillus anthracis*, *Bacillus cereus*, *Bacillus cereus* group, *Bacillus paranthracis*, *Bacillus thuringiensis*, *Bacillus wiedmannii*, food safety, foodborne illness, microbial source tracking, whole-genome sequencing (WGS)

## Abstract

Members of the Bacillus cereus sensu lato species complex, also known as the B. cereus group, vary in their ability to cause illness but are frequently isolated from foods, including meat products; however, food safety surveillance efforts that use whole-genome sequencing (WGS) often neglect these potential pathogens. Here, we evaluate the surveillance and source tracking potential of WGS as applied to B. cereus sensu lato by (i) using WGS to characterize B. cereus sensu lato strains isolated during routine surveillance of meat products across South Africa (*n *= 25) and (ii) comparing the genomes sequenced here to all publicly available, high-quality B. cereus sensu lato genomes (*n *= 2,887 total genomes). Strains sequenced here were collected from meat products obtained from (i) retail outlets, processing plants, and butcheries across six South African provinces (*n *= 23) and (ii) imports held at port of entry (*n *= 2). The 25 strains sequenced here were partitioned into 15 lineages via *in silico* seven-gene multilocus sequence typing (MLST). While none of the South African B. cereus sensu lato strains sequenced here were identical to publicly available genomes, six MLST lineages contained multiple strains sequenced in this study, which were identical or nearly identical at the whole-genome scale (≤3 core single nucleotide polymorphisms). Five MLST lineages contained (nearly) identical genomes collected from two or three South African provinces; one MLST lineage contained nearly identical genomes from two countries (South Africa and the Netherlands), indicating that B. cereus sensu lato can spread intra- and internationally via foodstuffs.

**IMPORTANCE** Nationwide foodborne pathogen surveillance programs that use high-resolution genomic methods have been shown to provide vast public health and economic benefits. However, Bacillus cereus sensu lato is often overlooked during large-scale routine WGS efforts. Thus, to our knowledge, no studies to date have evaluated the potential utility of WGS for B. cereus sensu lato surveillance and source tracking in foodstuffs. In this preliminary proof-of-concept study, we applied WGS to B. cereus sensu lato strains collected via South Africa’s national surveillance program of domestic and imported meat products, and we provide strong evidence that B. cereus sensu lato can be disseminated intra- and internationally via the agro-food supply chain. Our results showcase that WGS has the potential to be used for source tracking of B. cereus sensu lato in foods, although future WGS and metadata collection efforts are needed to ensure that B. cereus sensu lato surveillance initiatives are on par with those of other foodborne pathogens.

## INTRODUCTION

Bacillus cereus sensu lato, also known as the B. cereus group, is a complex of closely related, Gram-positive, spore-forming species, which are widespread throughout the environment (1). While some members of B. cereus sensu lato have important industrial applications or roles (e.g., as biocontrol agents in agricultural settings or as food spoilage organisms) (2–6), others are capable of causing illnesses or death in humans and/or animals (1, 7–9). Illnesses caused by members of B. cereus sensu lato can range in severity from mild to severe/fatal and include anthrax and anthrax-like illness (8, 10, 11), foodborne emetic intoxication (1, 7, 12–15), foodborne diarrheal toxicoinfection (1, 7, 14, 15), and nongastrointestinal infections (16, 17). As a foodborne pathogen, B. cereus is estimated to be responsible for more than 256,000 illnesses globally each year (18), although this is likely an underestimate due to the relatively mild and self-limiting nature of the symptoms that often accompany foodborne illness caused by B. cereus sensu lato (1).

Food safety surveillance efforts around the world have identified B. cereus sensu lato strains in a wide variety of foodstuffs (1, 7), including raw intact, processed, and ready-to-eat (RTE) meat and poultry products (19–27). In South Africa specifically, previous surveillance efforts have identified B. cereus sensu lato in (i) retail meats sold at supermarkets in the Pretoria area (i.e., Vienna sausages, salami, and poultry) (27) and (ii) biltong (a South African spiced intermediate moisture RTE meat product) sold at supermarkets, stalls, kiosks, and butcheries in Bloemfontein, Free State (28). Most recently, in a study of over 2,000 meat product samples collected from butcheries, processing plants, abattoirs, and retail outlets across all nine South African provinces, B. cereus sensu lato was present in 4.5 and 2.7% of domestic and imported meat products, respectively (26).

Ongoing surveillance efforts in South Africa have indicated that meat and poultry products can harbor B. cereus sensu lato and may pose a potential food safety risk to South African consumers. However, it is unclear which B. cereus sensu lato lineages are present in South African meat and poultry products on a genomic scale. Here, we used whole-genome sequencing (WGS) to characterize 25 B. cereus sensu lato strains isolated from raw intact, processed, and RTE meat and poultry products collected from processing plants, butcheries, and retail outlets across South Africa as well as imported meat products tested for B. cereus sensu lato at port of entry. By comparing South African strains sequenced here to all publicly available B. cereus sensu lato genomes (*n *= 2,887 total genomes), we identified multiple B. cereus sensu lato species present among South African meat and poultry products, and we detected multiple potential international and interprovincial B. cereus sensu lato dissemination events. Overall, our proof-of-concept study serves as the first genome-scale study of South African B. cereus sensu lato in foodstuffs and showcases the utility of WGS for B. cereus sensu lato surveillance and source tracking.

## RESULTS

### Multiple species are present among B. cereus sensu lato from South African meat and poultry products.

**FIG 1:**
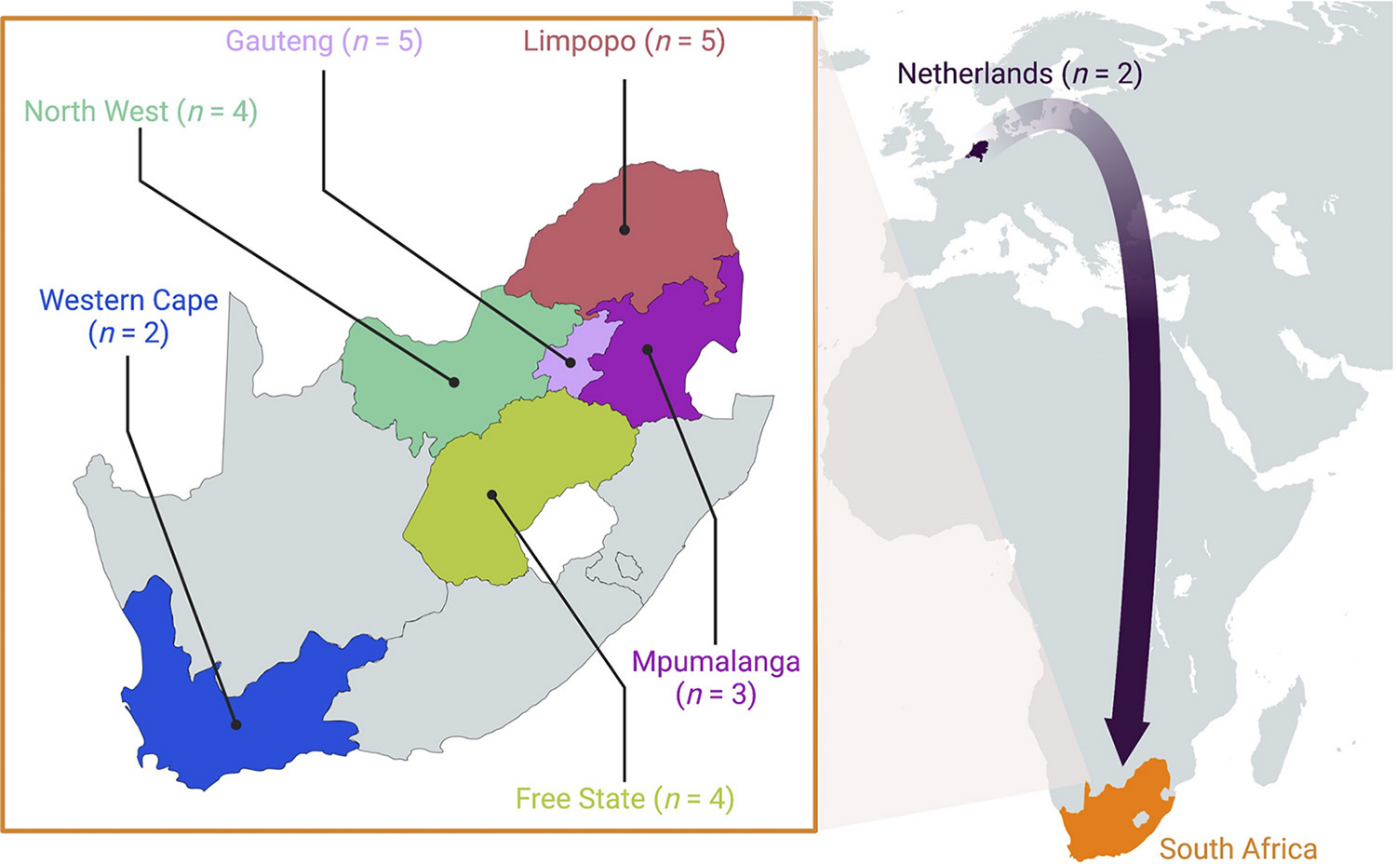
Geographic origins of B. cereus sensu lato strains sequenced in this study (*n *= 25). Strains affiliated with the Netherlands (*n *= 2) were isolated within South African borders at port of entry; however, the poultry products from which they were isolated were confirmed to originate from the Netherlands.

A total of 25 B. cereus sensu lato strains that had been isolated from meat and poultry products collected across South Africa in 2015 and 2016 (26) underwent WGS (Fig. 1, Table 1, and Table S1 in the supplemental material). Overall, 19 and 5 strains originated from beef and poultry products, respectively (76.0 and 20.0%, respectively), and 1 strain (4.0%) had been isolated from mixed-meat wors (a processed South African sausage; Table 1 and Table S1). The majority of strains (23 of 25, 92.0%) were obtained from domestic meat and poultry products acquired across six South African provinces (Fig. 1, Table 1, and Table S1). The remaining two strains (8.0%) were isolated from raw, intact chicken quarter legs, which had been imported into South Africa from the Netherlands and tested for the presence of B. cereus sensu lato at port of entry (Fig. 1, Table 1, and Table S1).

**TABLE 1:** B. cereus sensu lato strains sequenced in this study (*n *= 25)

Strain	Yr^*a*^	Meat sample type	Meat category^*b*^	Food animal	Establishment	Province^*c*^	*panC*^*d*^ group	MLST ST^*e*^	GTDB species^*f*^	2020 GSB species^*g*^
S57	2015	Beef biltong	RTE	Beef	Retail outlets	Free State	II	794	*wiedmannii*	*mosaicus*
S58	2015	Chicken thigh	Raw intact	Poultry	Retail outlets	Mpumalanga	III	NA	*anthracis* ^*h*^	*mosaicus*
S59	2016	Chicken quarter leg	Raw intact	Poultry	Cold store	Import (NL)	III	2413	*paranthracis*	*mosaicus*
S62	2015	Chicken thigh	Raw intact	Poultry	Retail outlets	Mpumalanga	III	2413	*paranthracis*	*mosaicus*
S64	2016	Sausage emulsion	RTE	Beef	Retail outlets	Western Cape	III	NA	*anthracis* ^*h*^	*mosaicus*
S66	2015	Beef mince	Processed	Beef	Processing plant	Mpumalanga	III	26	*paranthracis*	*mosaicus* subsp. *cereus*
S51	2015	Beef mince	Processed	Beef	Retail outlets	Limpopo	IV	1578	*thuringiensis*	*cereus sensu stricto*
S53	2015	Beef patties	Processed	Beef	Retail outlets	Gauteng	IV	2668	*thuringiensis_S*	*cereus sensu stricto*
S55	2015	Beef wors	Processed	Beef	Processing plant	Limpopo	IV	177	*cereus*	*cereus sensu stricto*
S56	2015	Beef wors	Processed	Beef	Processing plant	North West	IV	2721	*bombysepticus*	*cereus sensu stricto*
S63	2015	Beef biltong	Processed	Beef	Retail outlets	Free State	IV	2668	*thuringiensis_S*	*cereus sensu stricto*
S65	2015	Beef wors	Processed	Beef	Processing plant	Limpopo	IV	NA	*bombysepticus*	*cereus sensu stricto*
S67	2016	Chicken quarter leg	Raw intact	Poultry	Cold store	Import (NL)	IV	NA	*bombysepticus*	*cereus sensu stricto*
S70	2015	Beef mince	Processed	Beef	Retail outlets	Limpopo	IV	2668	*thuringiensis_S*	*cereus sensu stricto*
S77	2015	Beef wors	Processed	Beef	Retail outlets	Gauteng	IV	24	*cereus*	*cereus sensu stricto*
S78	2015	Beef biltong	RTE	Beef	Retail outlets	Gauteng	IV	1697	*thuringiensis_S*	*cereus sensu stricto*
S79	2015	Beef biltong	RTE	Beef	Retail outlets	North West	IV	2721	*bombysepticus*	*cereus sensu stricto*
S80	2015	Beef wors	Processed	Beef	Processing plant	North West	IV	177	*cereus*	*cereus sensu stricto*
S81	2016	Beef wors	Processed	Beef	Processing plant	North West	IV	NA	*cereus*	*cereus sensu stricto*
New_S84	2015	Beef patties	Processed	Beef	Retail outlets	Free State	IV	2721	*bombysepticus*	*cereus sensu stricto*
S85	2015	Beef wors	Processed	Beef	Retail outlets	Gauteng	IV	NA	*bombysepticus*	*cereus sensu stricto*
S86	2015	Sausage emulsion	RTE	Beef	Retail outlets	Western Cape	IV	2289	*cereus*	*cereus sensu stricto*
S87	2015	Frankfurter	Raw intact	Poultry	Processing plant	Free State	IV	NA	*bombysepticus*	*cereus sensu stricto*
S88	2015	Beef biltong	RTE	Beef	Retail outlets	Limpopo	IV	NA	*cereus*	*cereus sensu stricto*
S72	2015	Beef-pork-lamb wors	Processed	Mixed	Butchery	Gauteng	V	223	*toyonensis*	*toyonensis*

a Year of isolation.

b RTE, ready to eat.

c Two strains were isolated from meat products imported from the Netherlands (NL).

d *panC* phylogenetic group assigned using BTyper3.

e Sequence type (ST) assigned using the PubMLST seven-gene multilocus sequence typing (MLST) scheme for B. cereus and BTyper3; NA, not available.

f Genome Taxonomy Database (GTDB) species assigned using GTDB-Tk.

g Species and subspecies (where applicable) assigned using the 2020 genomospecies-subspecies-biovar (GSB) nomenclatural framework for B. cereus sensu lato and BTyper3 (see Table 2 for predicted biovar/phenotypic information).

h Despite GTDB assigning a species label of “B. anthracis,” these strains cannot cause anthrax illness nor do they belong to the classic “B. anthracis” lineage most commonly responsible for anthrax illness (34).

**FIG 2:**
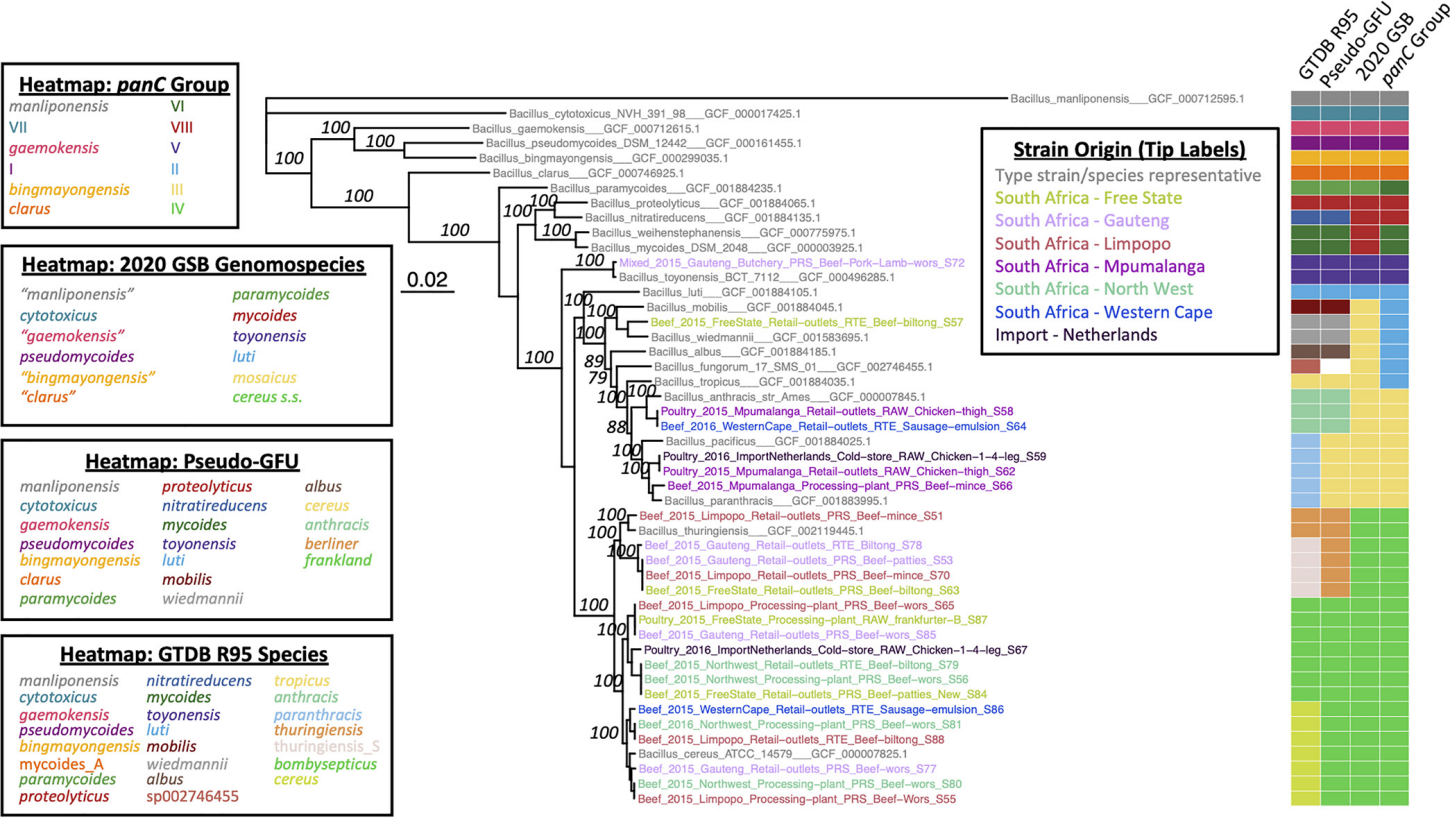
Maximum likelihood (ML) phylogeny constructed using amino acid sequences derived from the 25 B. cereus sensu lato isolate genomes sequenced in this study (tip labels colored by geographic origin) and type strain/species representative genomes of 23 published and effective B. cereus sensu lato species (gray tip labels). The heat map to the right of the phylogeny showcases species assignments obtained within the following taxonomic frameworks (from left to right): (i) genome taxonomy database (GTDB) release 05-RS95 and GTDB-Tk (GTDB R95), (ii) pseudo-gene flow units (GFUs) assigned using BTyper3 (pseudo-GFU), (iii) genomospecies of the 2020 standardized B. cereus sensu lato genomospecies-subspecies-biovar (GSB) framework and BTyper3 (2020 GSB), and (iv) *panC* group (I to VIII) assigned using BTyper3 (*panC* group). The phylogeny was constructed using IQ-TREE using core orthologs identified among all genomes via OrthoFinder as input. Branch lengths are reported in substitutions per site. Branch labels correspond to branch support percentages obtained using 1,000 replicates of the ultrafast bootstrap approximation (selected for readability). The type strain genome of effective B. cereus sensu lato species “*B. manliponensis*” (the most distant recognized member of B. cereus sensu lato) was used to root the phylogeny. Heat map legends for all four taxonomies are colored by their order of appearance in the heat map, from top to bottom; white heatmap cells denote genomes that could not be assigned to a taxonomic unit within a given taxonomic framework.

Species-level taxonomic classification of B. cereus sensu lato is notoriously challenging (1, 29, 30); to avoid taxonomic ambiguities and maximize interpretability, we applied multiple taxonomic assignment and sequence typing methods to the 25 strains sequenced here (Table 1). One such sequence typing framework relies on the pantoate-β-alanine ligase gene (*panC*) to assign B. cereus sensu lato strains to one of seven or more major phylogenetic groups, which have been proposed to conceptually serve as “species” (31, 32). Using the adjusted eight-group *panC* typing approach implemented in BTyper3 (33), the 25 B. cereus sensu lato strains sequenced here encompassed four *panC* phylogenetic groups (i.e., “species”; Fig. 2 and Table 1). The majority (*n *= 18 of 25, 72.0%) of the strains sequenced here were assigned to *panC* group IV (Fig. 2 and Table 1). The remaining strains were assigned to *panC* groups III, II, and V (*n *= 5, 1, and 1 strains, representing 20.0%, 4.0%, and 4.0% of isolates sequenced here, respectively; Fig. 2 and Table 1).

Using the Genome Taxonomy Database (GTDB) taxonomy, the 25 B. cereus sensu lato strains sequenced here encompassed eight genomospecies (Fig. 2 and Table 1). The 18 *panC* group IV strains sequenced here encompassed four GTDB genomospecies, while the five *panC* group III strains spanned two GTDB genomospecies (Fig. 2 and Table 1). The *panC* group II and group V strains (*n *= 1 each) were each assigned to separate GTDB genomospecies (Fig. 2 and Table 1).

Using a standardized genomospecies-subspecies-biovar (GSB) nomenclatural framework proposed for B. cereus sensu lato in 2020 (34) (referred to here as the “2020 GSB” framework), the 25 strains sequenced here encompassed three genomospecies (Fig. 2 and Tables 1 and 2). All 18 *panC* group IV strains were assigned to the B. cereus sensu stricto genomospecies (Fig. 2 and Tables 1 and 2); genes encoding insecticidal toxins (referred to here as “Bt toxin-encoding genes”) were detected within all group IV B. cereus sensu stricto genomes (using BtToxin_scanner2’s “old” gene detection approach), meaning that these 18 strains were predicted to belong to the Thuringiensis biovar (i.e., B. cereus sensu stricto bv. Thuringiensis; Table 2). All five *panC* group III and the single *panC* group II strain(s) were assigned to genomospecies *B. mosaicus* within the 2020 GSB framework (Fig. 2 and Tables 1 and 2). One *panC* group III *B. mosaicus* strain (i.e., strain S66) was assigned to PubMLST sequence type 26 (ST26) and possessed cereulide (emetic toxin) synthetase-encoding *cesABCD* and was thus assigned to the *cereus* subspecies and biovar Emeticus (i.e., *B. mosaicus* subsp. *cereus* bv. Emeticus; Table 2). The lone *panC* group V strain sequenced here was assigned to the *B. toyonensis* genomospecies; Bt toxin-encoding genes were detected in this genome (via BtToxin_scanner2’s “old” gene detection approach), and thus this strain was predicted to belong to biovar Thuringiensis (i.e., *B. toyonensis* bv. Thuringiensis; Fig. 2 and Tables 1 and 2).

**TABLE 2:** Predicted phenotypes of B. cereus sensu lato strains sequenced in this study (*n *= 25)

Strain	*panC*^*a*^ group	MLST ST^*b*^	GTDB species^*c*^	Anthrax toxin and capsule genes^*d*^^,^^*e*^	Emetic toxin *cesABCD*^*d*^	Diarrheal toxin *nheABC*^*d*^	Diarrheal toxin *hblACD*^*d*^	Diarrheal toxin *cytK-2*^*d*^	Bt toxin genes^*f*^	2020 GSB taxonomy^*g*^^,^^*h*^
S57	II	794	*wiedmannii*	−	−	+	+	+	−/−/+	*B. mosaicus*
S58	III	NA	*anthracis* ^*i*^	−	−	+	−	+	−/−/+	
S59	III	2413	*paranthracis*	−	−	+	−	−	−/−/−	
S62	III	2413	*paranthracis*	−	−	+	−	−	−/−/+	
S64	III	NA	*anthracis* ^*i*^	−	−	+	−	+	−/−/+	
S66	III	26	*paranthracis*	−	+	+	−	−	−/−/+	*B. mosaicus* subsp. *cereus* bv. Emeticus; B. cereus bv. Emeticus; *B.* Emeticus
S51	IV	1578	*thuringiensis*	−	−	+	+	+	−/+/+	B. cereus sensu stricto bv. Thuringiensis; Thuringiensis
S53	IV	2668	*thuringiensis_S*	−	−	+	+	+	−/+/−	
S55	IV	177	*cereus*	−	−	+	+	+	−/+/+	
S56	IV	2721	*bombysepticus*	−	−	+	+	+	−/+/−	
S63	IV	2668	*thuringiensis_S*	−	−	+	+	+	−/+/+	
S65	IV	NA	*bombysepticus*	−	−	+	+	−	+/+/−	
S67	IV	NA	*bombysepticus*	−	−	+	+	+	−/+/+	
S70	IV	2668	*thuringiensis_S*	−	−	+	+	+	−/+/−	
S77	IV	24	*cereus*	−	−	+	+	+	−/+/+	
S78	IV	1697	*thuringiensis_S*	−	−	+	+	−	+/+/+	
S79	IV	2721	*bombysepticus*	−	−	+	+	+	−/+/−	
S80	IV	177	*cereus*	−	−	+	+	+	−/+/+	
S81	IV	NA	*cereus*	−	−	+	+	+	−/+/−	
New_S84	IV	2721	*bombysepticus*	−	−	+	+	+	−/+/+	
S85	IV	NA	*bombysepticus*	−	−	+	+	−	+/+/+	
S86	IV	2289	*cereus*	−	−	+	+	+	−/+/+	
S87	IV	NA	*bombysepticus*	−	−	+	+	−	+/+/−	
S88	IV	NA	*cereus*	−	−	+	+	+	−/+/+	
S72	V	223	*toyonensis*	−	−	+	+	−^*j*^	−/+/+	*B. toyonensis* bv. Thuringiensis; Thuringiensis

a *panC* phylogenetic group assigned using BTyper3.

b Sequence type (ST) assigned using the PubMLST seven-gene multilocus sequence typing (MLST) scheme for B. cereus and BTyper3; NA, not available.

c Genome Taxonomy Database (GTDB) species assigned using GTDB-Tk.

d Viurlence factors detected in each genome using BTyper3 and default thresholds (70% amino acid identity and 80% coverage); presence and absence of virulence factors are denoted by “+” and “−”, respectively.

e All of *cya*, *lef*, *pagA* (toxin), *capABCDE*, *hasABC*, and/or *bpsXABCDEFGH* (capsules).

f B. thuringiensis (Bt) insecticidal toxin-encoding genes detected using (i) BTyper3 (which uses a conservative BLAST-based approach and minimum default amino acid identity and coverage thresholds of 50 and 70%, respectively) and BtToxin_scanner2’s (ii) “old” and (iii) “new” gene detection approaches (detected using default thresholds), each separated by a solidus (“/”).

g Species, subspecies (where applicable), and biovar (where applicable) assigned using the 2020 genomospecies-subspecies-biovar (GSB) nomenclatural framework for B. cereus sensu lato and BTyper3; multiple taxonomic labels are listed for strains that can be referenced using shorted subspecies and/or biovar notation (separated by a semicolon).

h Biovar Thuringiensis was assigned to genomes in which Bt insecticidal toxin-encoding genes were detected using BtToxin_scanner2’s “old” gene detection approach and default settings (which is less conservative than BTyper3’s Bt toxin gene detection approach).

i Despite GTDB assigning a species label of “B. anthracis,” these strains cannot cause anthrax illness nor do they belong to the classic “B. anthracis” lineage most commonly responsible for anthrax illness (34).

j The gene was present when the minimum coverage threshold was lowered to 0%.

Using a rapid, average nucleotide identity (ANI)-based pseudo-gene flow unit (GFU) assignment scheme, which attempts to assign B. cereus sensu lato genomes to taxonomic units that mimic B. cereus sensu lato “species” previously delineated using recent gene flow (33), the 25 strains sequenced here were assigned to six pseudo-GFUs (Fig. 2 and Table S1). The 18 *panC* group IV and five *panC* group III strains sequenced here each spanned two pseudo-GFUs (Fig. 2 and Table S1). The *panC* group II and group V strains (*n *= 1 each) were each assigned to separate pseudo-GFUs, respectively (Fig. 2 and Table S1).

As mentioned above, considerable phenotypic diversity was predicted among strains sequenced here; one strain harbored cereulide synthetase-encoding genes, and 19 strains possessed Bt toxin-encoding genes (detected using BtToxin_scanner2’s “old” gene detection approach; Table 2), although the toxin production and insecticidal capabilities of the strains sequenced here were not evaluated *in vitro* or *in vivo*. No anthrax toxin- or capsule-encoding genes were identified within the genomes of the isolates sequenced here (Table 2).

Overall, regardless of whether the *panC*, GTDB, 2020 GSB, or pseudo-GFU assignment frameworks were used, B. cereus sensu lato strains isolated from meat and poultry products in South Africa were considerably diverse and represented multiple genomospecies (Fig. 2; Tables 1 and 2; Table S1). Additionally, using PubMLST’s seven-gene MLST scheme for B. cereus, 17 of 25 strains (68.0%) encompassed 11 STs, with eight strains (32.0%) assigned to unknown STs (Table 1 and Table S1). Due to the considerable genomic diversity observed among isolates sequenced here, major lineages represented by strains sequenced in this study are discussed individually in detail below, largely within the context of *panC* groups and/or MLST STs, as these frameworks are well established (31, 32, 35, 36) and likely the most interpretable to readers.

### Several B. cereus sensu lato lineages within *panC* group IV are distributed across multiple South African provinces.

**FIG 3:**
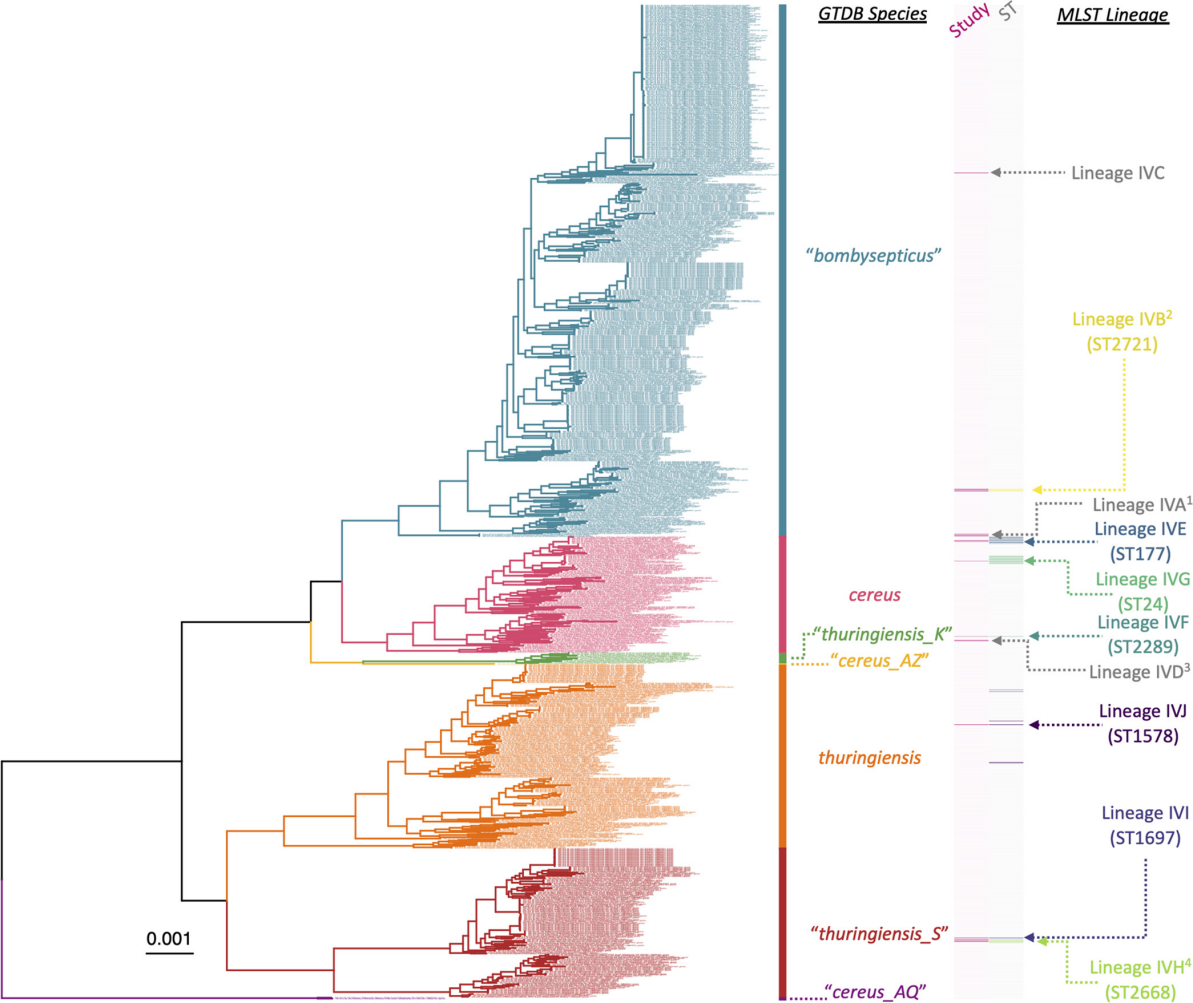
Maximum likelihood (ML) phylogeny constructed using core SNPs identified among orthologous gene clusters of 1,081 *panC* group IV B. cereus sensu lato genomes. The phylogeny was rooted using *panC* group III B. anthracis strain Ames Ancestor as an outgroup (NCBI RefSeq accession number GCF_000008445.1; omitted for readability). Tip label colors and clade labels denote species assigned using GTDB-Tk (“GTDB Species”). The heat map to the right of the phylogeny denotes (i) whether a strain was sequenced in this study (dark pink) or not (light pink; “Study”) and (ii) multilocus sequence typing (MLST) sequence types (STs) associated with strains sequenced in this study, where applicable (colored), or not (gray; “ST”). MLST lineages discussed in Table 3 are annotated to the right of the heat map (“MLST Lineage”). MLST lineages with numerical superscripts contain two or more strains sequenced in this study, which were highly similar on a genomic scale; these lineages are depicted in Fig. 4. Branch lengths are reported in substitutions per site.

**FIG 4:**
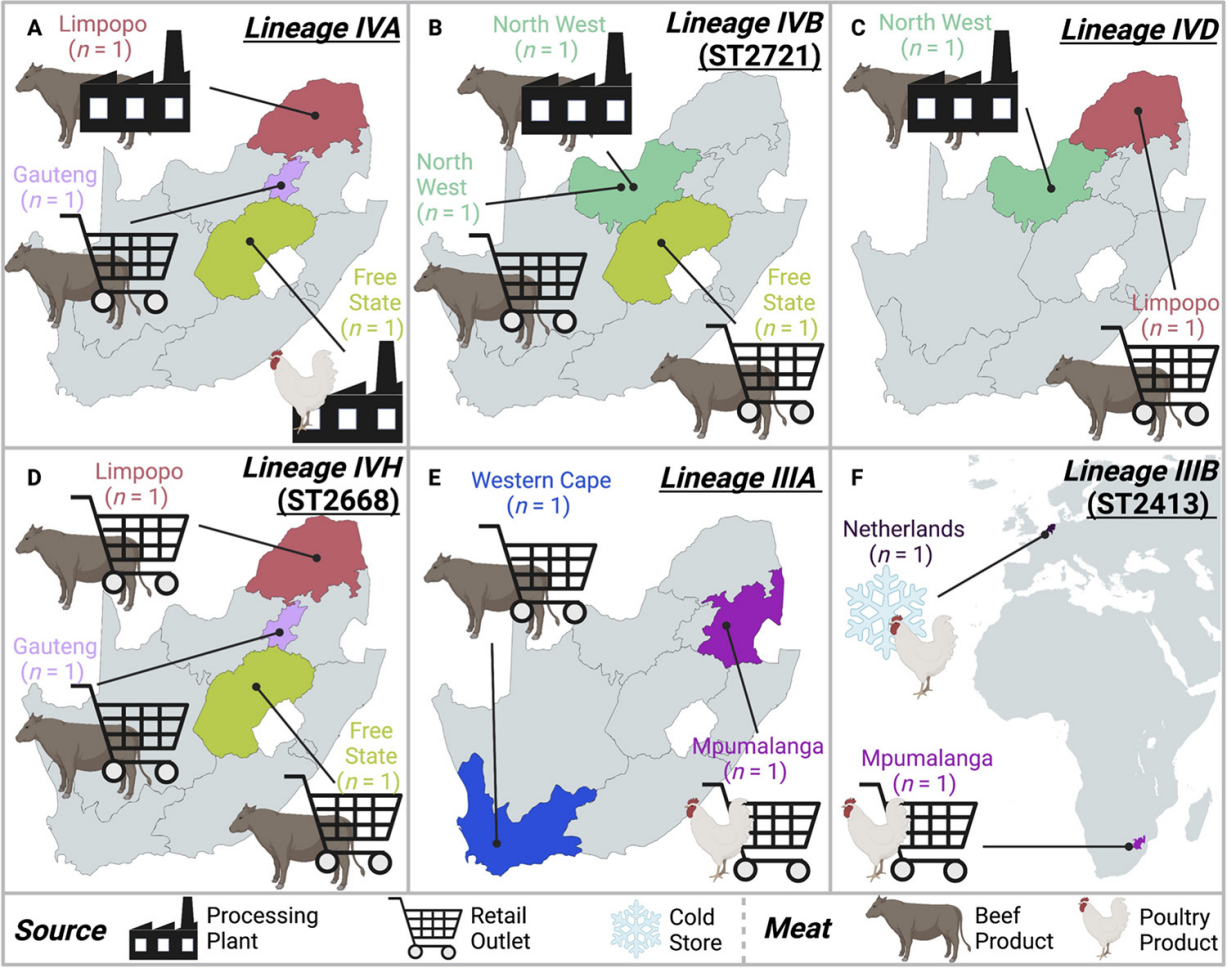
B. cereus sensu lato multilocus sequence typing (MLST) lineages that contained two or more strains sequenced in this study, which were identical or nearly identical at the whole-genome scale (pairwise core single nucleotide polymorphism [SNP] differences of ≤3). Lineage names and sequence types (STs), where applicable, are shown in the top right corner. Geographic and source origins of each strain are displayed in the respective map. Strains affiliated with the Netherlands (*n *= 2) were isolated within South African borders; however, the poultry products from which they were isolated were confirmed to originate from the Netherlands.

Within *panC* group IV, the 18 strains sequenced here were partitioned into 10 lineages using MLST (referred to here as “MLST lineages”; Table 3). Based on (i) whole-genome phylogenies, (ii) pairwise core single nucleotide polymorphisms (SNPs) identified within MLST lineages, and (iii) ANI values calculated within and between MLST lineages, 4 of 10 *panC* group IV MLST lineages contained South African strains sequenced in this study, which were highly similar to at least one other strain at the whole-genome level (Fig. 3 and 4 and Table 3). One of these lineages (denoted in Table 3 as lineage IVA) was composed of three South African strains sequenced in this study (S65, S85, and S87), which were assigned to GTDB’s “*B. bombysepticus*” genomospecies and belonged to an unknown ST (Fig. 3 and 4 and Table 3). Despite all three genomes being nearly identical (pairwise core SNP distance of 0, pairwise ANI of >99.99; Fig. 3 and Table 3), the three strains were isolated from (i) three different establishments and provinces (a processing plant in Limpopo, a retail outlet in Gauteng, and a processing plant in Free State) and (ii) two different types of meat products (two strains from beef wors and one from a poultry frankfurter; Fig. 4 and Table 1).

**TABLE 3:** Genomic distances within multilocus sequence typing (MLST) lineages, which contain strains sequenced in this study

			No. of genomes				Pairwise SNP range within MLST lineage		
MLST lineage^*a*^	GTDB species^*b*^	MLST ST^*c*^	Total	Study	ANI range (mean)^*d*^	Reference genome^*e*^	Total (mean)^*f*^	Within-study (mean)^*g*^	Study-public (mean)^*h*^
IVA*	*bombysepticus*	NA	3	3	99.99–100.0 (100.0)	S85	0–0 (0)	0-0 (0)	NA
IVB*	*bombysepticus*	2721	3	3	99.99–100.0 (100.0)	S79	0–0 (0)	0-0 (0)	NA
IVC	*bombysepticus*	NA	1	1	NA	NA	NA	NA	NA
IVD*	*cereus*	NA	2	2	100.0–100.0 (100.0)	S81	3	3	NA
IVE	*cereus*	177	11	2	99.70–100.0 (99.92)	S80	1–178 (60.18)	16	10–157 (48.56)
IVF	*cereus*	2289	1	1	NA	NA	NA	NA	NA
IVG	*cereus*	24^*i*^^,^^*j*^	12	1	99.75–100.0 (99.91)	S77	1–910 (298.2)	NA	94–889 (272.8)
IVH*	*thuringiensis_S*	2668	4	3	98.90–100.0 (99.49)	S53	0–23,098 (11,549)	0-1 (0.67)	23,097–23,098 (23,097)
IVI	*thuringiensis_S*	1697	1	1	NA	NA	NA	NA	NA
IVJ	*thuringiensis*	1578^*j*^	6	1	98.49–99.92 (98.81)	S51	245–8,636 (6,151)	NA	1,865-8,353 (5,885)
IIA	*wiedmannii*	794	3	1	99.95–100.0 (99.97)	S57	31–105 (78.67)	NA	31–100 (65.50)
IIIA*	*anthracis* ^*k*^	NA	2	2	99.84–100.0 (99.92)	S64	0	0	NA
IIIB*	*paranthracis*	2413	2	2	100.0–100.0 (100.0)	S59	1	1	NA
IIIC	*paranthracis*	26	77	1	99.52–100.0 (99.84)	S66	480 (159.5)	NA	85–361(139.2)
VA	*toyonensis*	223^*j*^	41	1	98.95–99.99 (99.51)	S72	5–1,767 (991.1)	NA	23–1,684 (746.4)

a Lineage identifiers (IDs) pertain to clusters displayed in Fig. 3 (group IV), Fig. 5 (groups II and III), and Fig. 6 (group V); IDs with an asterisk contain two or more strains sequenced in this study, which were highly similar on a genomic scale.

b Genome Taxonomy Database (GTDB) species assigned using GTDB-Tk.

c Sequence type (ST) assigned using the PubMLST seven-gene MLST scheme for B. cereus and BTyper3; NA, not available.

d FastANI average nucleotide identity (ANI) range and mean values between all genomes in the lineage (excludes self-comparisons); NA, not available/applicable.

e Strain sequenced in this study, which was used as a reference genome for single nucleotide polymorphism (SNP) calling among all genomes within the lineage via Snippy; NA, not available/applicable.

f Pairwise SNP distances calculated among all genomes within the lineage (excludes self-comparisons); NA, not available/applicable.

g Pairwise SNP distances calculated among all genomes within the lineage that were sequenced in this study (excludes self-comparisons); NA, not available/applicable.

h Pairwise SNP distances calculated between genomes sequenced in this study and public genomes within the same lineage (excludes self-comparisons); NA, not available/applicable.

i For polyphyletic ST24, one outlier ST24 genome was excluded (NCBI RefSeq accession number GCF_010580595.1).

j ST was polyphyletic.

k Despite GTDB assigning a species label of “B. anthracis,” these strains cannot cause anthrax illness nor do they belong to the classic “B. anthracis” lineage most commonly responsible for anthrax illness (34).

Similar results were observed for *panC* group IV ST2721 (lineage IVB in Table 3), which was also assigned to GTDB’s “*B. bombysepticus*” genomospecies and contained three strains sequenced in this study (S56, S79, and New_S84; Fig. 3 and 4 and Table 3). The three ST2721 genomes were nearly identical (pairwise core SNP distance of 0, pairwise ANI of >99.99; Table 3), despite the fact that the strains originated from three different meat products (one from each of beef wors, beef biltong, and processed beef patties) obtained from three different establishments (from a processing plant in North West province, a retail outlet in North West province, and a retail outlet in Free State, respectively; Fig. 4 and Table 1).

A third *panC* group IV lineage of unknown ST, which was assigned to GTDB’s B. cereus species (lineage IVD), contained two isolates sequenced in this study (S81 and S88; Fig. 3 and 4 and Table 3). One strain (S81) had been isolated from beef wors in a processing plant in the North West province; the other strain (S88) was from RTE beef biltong in a retail outlet in Limpopo (Fig. 4 and Table 1). Both strains were highly similar on a genomic scale (>99.99 ANI) and differed by three core SNPs (Fig. 3 and Table 3). For reference, in a previous point source foodborne outbreak caused by B. cereus sensu lato (37), outbreak isolates could differ by up to seven core SNPs (using the same SNP-calling methodology used here) (38).

A fourth *panC* group IV lineage, ST2668, contained three strains sequenced in this study (S53, S63, and S70), which were assigned to GTDB’s “B. thuringiensis*_S*” genomospecies (lineage IVH; Fig. 3 and 4 and Table 3). The three strains sequenced here differed by, at most, a single core SNP (Table 3), even though all had been isolated from different meat products (processed beef patties, beef biltong, and processed beef mince) obtained from different establishments (i.e., from retail outlets in each of Gauteng, Free State, and Limpopo, respectively; Fig. 4 and Table 1). One publicly available genome associated with a B. cereus sensu lato strain isolated from grass in KarieDeshe, Israel (39), was additionally assigned to ST2668 (NCBI RefSeq accession number GCF_005217245.1); however, this strain was not closely related to the three nearly identical South African ST2668 strains sequenced in this study (Table 3).

The remaining six MLST lineages (i.e., lineages IVC, IVE, IVF, IVG, IVI, and IVJ corresponding to an unknown ST, ST177, ST2289, ST24, ST1697, and ST1578, respectively; Table 3) contained isolates sequenced in this study, which were not closely related to any other strains at the whole-genome level (Fig. 3 and Table 3). Lineages IVC, IVF, and IVI were singleton lineages, which each contained one genome sequenced in this study (S67, S86, and S78, respectively), and shared 98.90 to 99.35 ANI with their closest publicly available neighbors (Fig. 3 and Table 3). Two lineages (IVG and IVJ) each contained multiple genomes, but only one genome sequenced in this study (i.e., S77 and S51, respectively; Fig. 3 and Table 3); for both lineages, the South African genome sequenced here was not identical to any publicly available genomes (≥94 core SNP distance; Table 3). The remaining lineage, lineage IVE (ST177), contained multiple genomes as well as multiple genomes sequenced in this study (Fig. 3 and Table 3). Notably, the two ST177 strains sequenced in this study (S55 and S80), which were assigned to this lineage, were not identical and were isolated from beef wors from processing plants in the Limpopo and North West provinces, respectively (Table 1), highlighting that WGS can be useful for differentiating closely related B. cereus sensu lato genomes within STs.

### A *panC* group III B. cereus sensu lato lineage with a novel sequence type was detected in beef and poultry products from two South African provinces.

Two *panC* group III B. cereus sensu lato strains sequenced here were assigned to an unknown ST (S58 and S64; Fig. 5 and Table 1). Despite having been isolated from different products (S58 from a raw chicken thigh and S64 from RTE sausage emulsion) from different establishments (retail outlets in Mpumalanga and Western Cape, respectively), the strains were nearly identical (pairwise core SNP distance of 0, pairwise ANI of >99.84; Fig. 4 and 5 and Table 3), indicating that these two strains may share a source.

**FIG 5:**
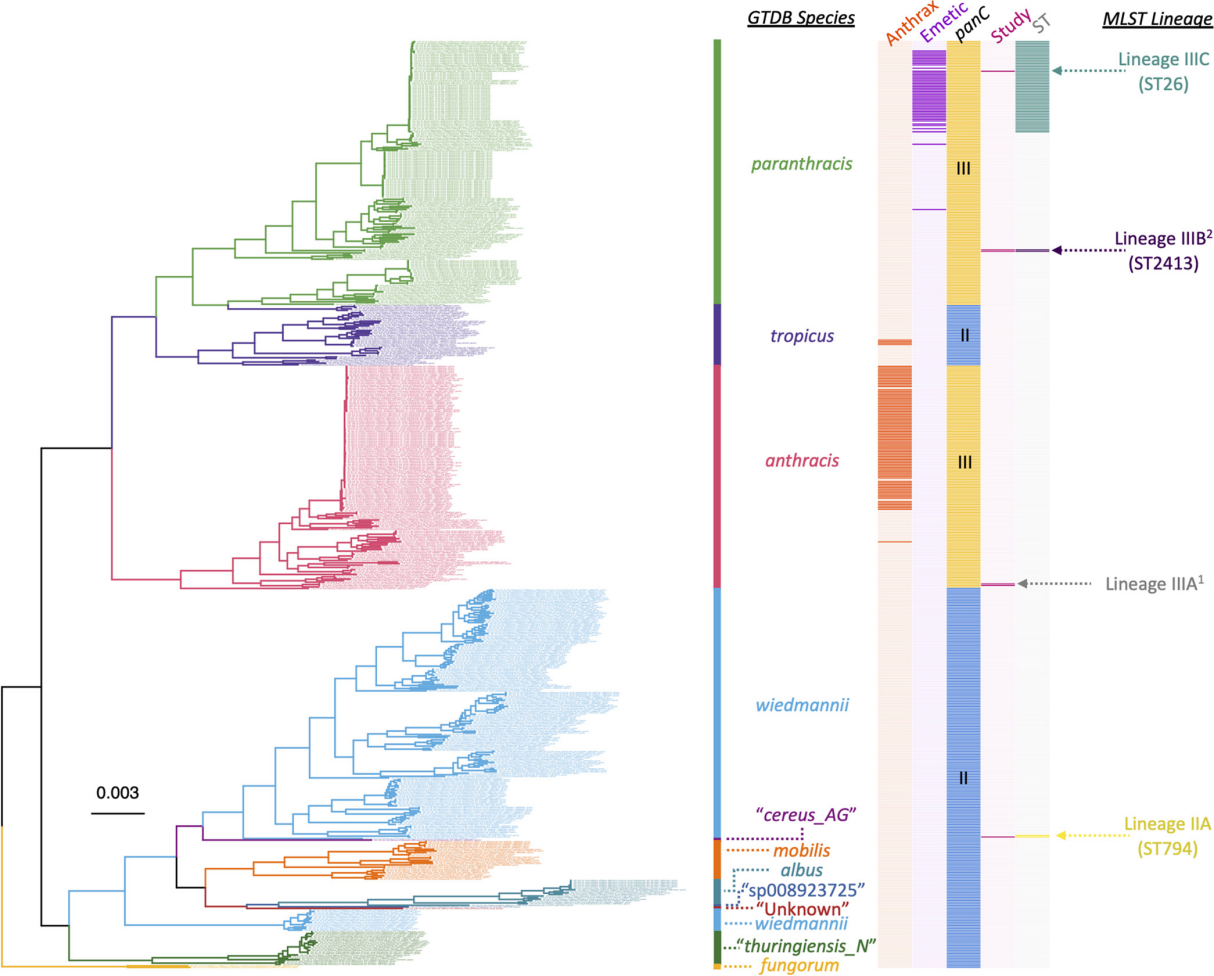
Maximum likelihood phylogeny constructed using core SNPs identified among orthologous gene clusters of 597 B. mosaicus (as defined within the 2020 genomospecies-subspecies-biovar [GSB] taxonomy). The phylogeny was rooted using *panC* group IV B. cereus strain ATCC 14579 as an outgroup (NCBI RefSeq accession number GCF_006094295.1; omitted for readability). Tip label colors and clade labels denote species assigned using GTDB-Tk (“GTDB Species”). The heat map to the right of the phylogeny denotes (i) whether a strain possesses anthrax toxin-encoding genes *cya*, *lef*, and *pagA* (dark orange) or not (light orange; “Anthrax”); (ii) whether a strain possesses cereulide synthetase-encoding *cesABCD* (dark purple) or not (light purple; “Emetic”); (iii) whether a strain belongs to *panC* group II (blue) or III (yellow; *“panC*”); (iv) whether a strain was sequenced in this study (dark pink) or not (light pink; “Study”); and (v) multilocus sequence typing (MLST) sequence types (STs) associated with strains sequenced in this study, where applicable (colored), or not (gray; “ST”). MLST lineages discussed in Table 3 are annotated to the right of the heatmap (“MLST Lineage”). MLST lineages with numerical superscripts contain two or more strains sequenced in this study, which were highly similar on a genomic scale; these lineages are depicted in Fig. 4. Branch lengths are reported in substitutions per site.

### A *panC* group III B. cereus sensu lato strain with cereulide synthetase-encoding genes belongs to the “high-risk” ST26 lineage.

One *panC* group III B. cereus sensu lato strain (S66) was assigned to GTDB’s *B. paranthracis* genomospecies and possessed cereulide synthetase-encoding genes (Fig. 5 and Table 2). This strain, which had been isolated from processed beef mince from a processing plant in Mpumalanga, was a member of ST26 (Fig. 5 and Table 2), the ST to which most cereulide-producing B. cereus sensu lato strains belong (although it should be noted that ST26 strains may be capable of producing enterotoxins and causing diarrheal illness regardless of whether they produce cereulide or not) (38, 40, 41). While members of ST26 are comparatively closely related (>99.52 pairwise ANI), WGS was able to distinguish the South African strain sequenced here from closely related ST26 strains (pairwise core SNP distance of ≥85 relative to publicly available genomes after removing recombination; Fig. 5 and Table 3).

### A *panC* group III B. cereus sensu lato lineage assigned to ST2413 shows evidence of intercontinental dissemination.

Two *panC* group III B. cereus sensu lato strains sequenced in this study (S59 and S62) were assigned to ST2413 within GTDB’s *B. paranthracis* species (Fig. 5 and Table 3). Unlike the ST26 strain, which was also assigned to GTDB’s *B. paranthracis* species, neither ST2413 strain possessed cereulide synthetase-encoding genes (Fig. 5 and Table 2). Both strains were isolated from raw chicken; however, S59 was isolated at port of entry from a chicken quarter leg that had been imported from the Netherlands, and S62 was isolated from a chicken thigh sold at a retail outlet in Mpumalanga (Fig. 4 and Table 1). Notably, these strains were nearly identical on a genomic scale (pairwise core SNP distance of 1, pairwise ANI of 100.0; Fig. 5 and Table 3), despite originating from different continents (i.e., Europe and Africa; Fig. 4 and Table 1).

### A *panC* group II B. cereus sensu lato strain assigned to ST794 is most closely related to a food-associated strain responsible for diarrheal illness in Norway.

One *panC* group II B. cereus sensu lato strain was sequenced in this study (S57) and was assigned to ST794 within GTDB’s *B. wiedmannii* species (Fig. 5 and Table 1). Strain S57 had been isolated from RTE beef biltong sold at a retail outlet in Free State (Table 1). Compared to the two publicly available ST794 genomes, S57 shared greater than 99.9 ANI with both publicly available genomes and differed by 31 and 100 core SNPs (relative to genomes with NCBI RefSeq assembly accession numbers GCF_900094845.1 and GCF_007671965.1, respectively; Fig. 5 and Table 3). Notably, beef biltong-associated strain S57 sequenced here was most closely related to strain NVH 0674-98, a psychrotolerant strain that had been isolated in Norway from mashed swedes, which were reportedly responsible for diarrheal foodborne illness (42). The other ST794 strain, DE0555, was an environmental isolate collected in 2018 from Durham, NC, in the United States (NCBI BioSample accession number SAMN11792715).

### A *panC* group V B. cereus sensu lato strain from South African mixed-meat wors most closely resembles a plant-associated strain from the United States.

**FIG 6:**
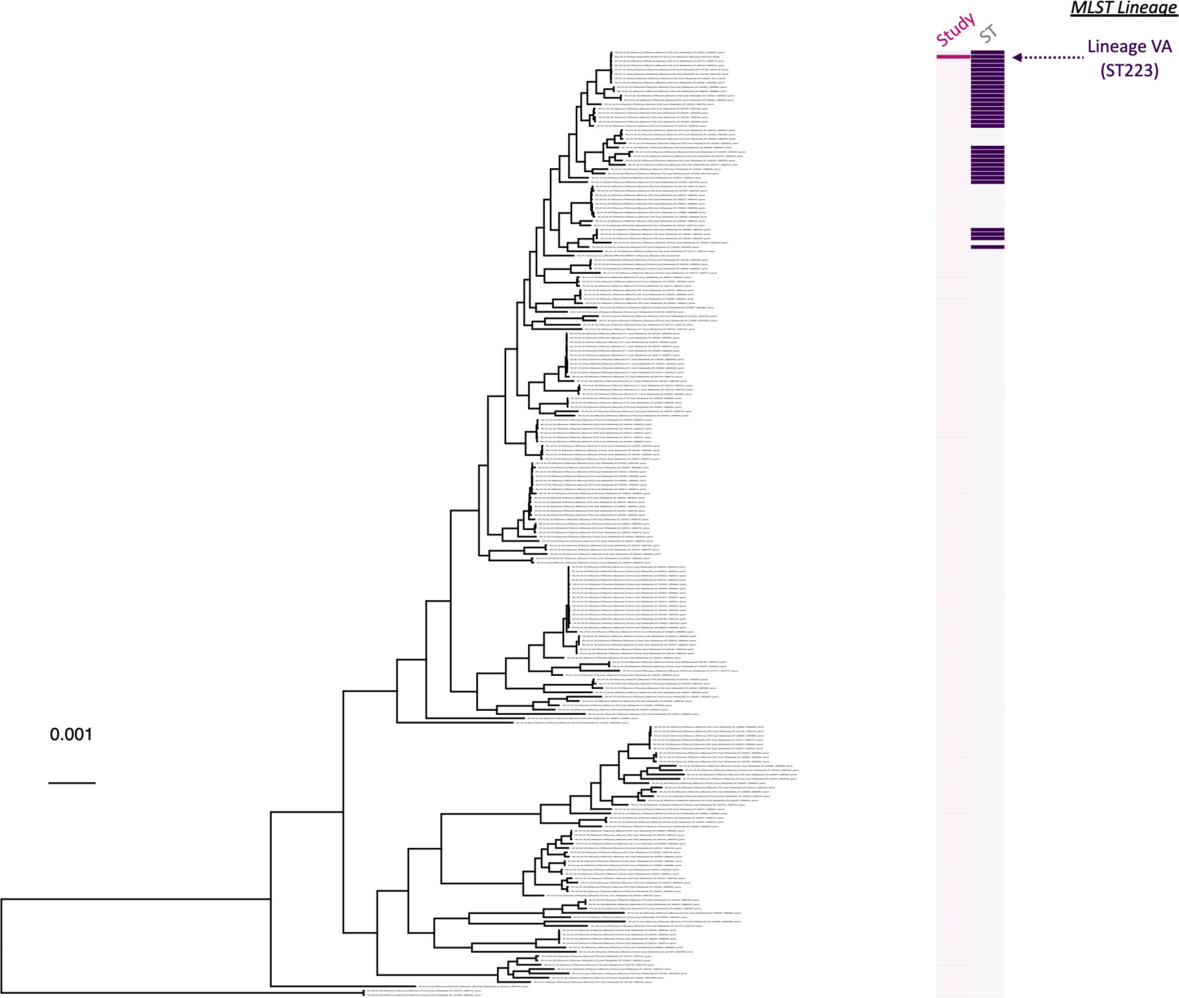
Maximum likelihood phylogeny constructed using core SNPs identified among orthologous gene clusters of 219 *panC* group V *B. toyonensis* genomes. The phylogeny was rooted using *panC* group IV B. cereus strain ATCC 14579 as an outgroup (NCBI RefSeq accession number GCF_006094295.1; omitted for readability). The heat map to the right of the phylogeny denotes (i) whether a strain was sequenced in this study (dark pink) or not (light pink; “Study”) and (ii) multilocus sequence typing (MLST) sequence types (STs) associated with strains sequenced in this study, where applicable (colored), or not (gray; “ST”). MLST lineages discussed in Table 3 are annotated to the right of the heat map. Branch lengths are reported in substitutions per site.

One *panC* group V B. cereus sensu lato strain was sequenced in this study (S72) and was assigned to ST223 within GTDB’s *B. toyonensis* species (Fig. 6 and Tables 1 to 3). S72 had been isolated in a butchery in Gauteng from a processed wors composed of a mix of beef, pork, and lamb (Table 1). Strain S72 was most closely related to a publicly available genome, strain AFS092321 (NCBI RefSeq accession number GCF_002552615.1), which had been isolated in 2014 from a tree leaf in North Carolina, United States (NCBI BioSample accession SAMN07598612) (43); the two strains shared greater than 99.9 ANI and differed by 23 core SNPs (Fig. 6 and Table 3).

## DISCUSSION

### B. cereus sensu lato lineages can be disseminated inter- and intranationally via the food supply chain.

The movement of commodities (e.g., foods, animals, animal products, agricultural products, and consumer products) through inter- and intranational trade can contribute to the global, regional, and local dissemination of microorganisms, including pathogens (44–48). The international agro-food trade specifically plays an increasingly pivotal role in providing food supplies to communities around the globe but can contribute to the dissemination of foodborne pathogens (47, 48). Consequently, high-resolution technologies, such as WGS, are being used increasingly to monitor the spread of pathogens along the food supply chain (49, 50).

Using WGS, we identified six South African B. cereus sensu lato lineages, which showcased evidence of interregional dissemination (Fig. 4 and Table 3). Notably, one B. cereus sensu lato lineage showed evidence of intercontinental spread between Europe and Africa. A B. cereus sensu lato ST2413 strain isolated from raw chicken sold in retail outlets in Mpumalanga, South Africa, was identical to a ST2413 strain isolated from chicken imported from the Netherlands and tested for B. cereus sensu lato at port of entry. We may hypothesize that the raw chicken sold in Mpumalanga originated from the Netherlands, as the Netherlands was the second-largest exporter of chicken meat products to South Africa in 2014 to 2016 (i.e., the time frame in which the strains sequenced here were collected) (51); however, this is merely a hypothesis, as we were unable to confirm this with the retail outlet, and no publicly available B. cereus sensu lato genomes from the Netherlands or elsewhere were closely related to the two ST2413 genomes collected here. Regardless, all B. cereus sensu lato strains isolated from imported meat products for this study were collected at port of entry. Imported food products are routinely inspected for the presence of foodborne pathogens before their entry into the country via the South African government’s national foodborne bacterial pathogen surveillance program. Thus, there is strong evidence that the B. cereus sensu lato strains collected from imported meat and poultry products and sequenced here originated from outside South Africa.

We additionally identified five B. cereus sensu lato lineages, which showed evidence of interprovincial spread within South Africa: four *panC* group IV lineages and one *panC* group III lineage were each composed of (nearly) identical strains, which were isolated from meat products in two or more South African provinces (Fig. 4 and Table 3). Thus, it is likely that strains within each lineage shared a common source; however, a lack of additional metadata and genomes prevents confirmation of this. Overall, these results showcase the utility of WGS for B. cereus sensu lato source tracking and surveillance, although future studies relying on additional genomes with detailed metadata are needed.

### Nomenclatural frameworks that incorporate both genomic and phenotypic data can improve strain-level B. cereus sensu lato risk assessment.

While B. cereus sensu lato strain isolation and culturing protocols (e.g., choice of medium and growth temperature) may preferentially select for or against some B. cereus sensu lato lineages (37, 52–54), multiple B. cereus sensu lato species were identified among strains isolated from South African meat products, regardless of the taxonomic framework used. B. cereus sensu lato species delineation is notoriously challenging, and numerous B. cereus sensu lato taxonomic frameworks have been proposed (29). Phenotypic traits historically used for B. cereus sensu lato species assignment (e.g., motility, colony morphology, and ability to cause illness) have long been known to be inconsistent with genome evolution (29, 31, 32). Taxonomies that rely solely on genomic data, however, may lead to incorrect assumptions of a strain’s pathogenic potential, particularly when taxonomic labels have deep roots in medicine or industry (29, 34).

For example, within some ANI-based taxonomic frameworks (e.g., GTDB), the B. anthracis genomospecies includes B. cereus sensu lato strains that, historically, would be referred to as “B. cereus” or “group III B. cereus”; these strains possess phenotypic characteristics associated with “B. cereus” (e.g., as outlined in the United States Food and Drug Administration’s Bacteriological Analytical Manual) (53, 54), and, like “B. cereus,” they are incapable of causing anthrax (34). These nonanthrax-causing group III B. cereus sensu lato strains have been isolated from diverse environments, including foods (e.g., milk, egg whites, and spices), consumer products (e.g., baby wipes), and soil, indicating that these organisms are not uncommon in environmental and industrial settings (34). Thus, as WGS becomes more popular in clinical and industrial settings, it is possible that professionals who rely solely on increasingly popular genomic methods for taxonomic delineation (e.g., GTDB, ANI-based comparisons to species type strains) may incorrectly assume that these strains can cause anthrax due to the “B. anthracis” species labels that some taxonomic classification programs produce (29, 34). Here, during routine surveillance of meat products in South Africa, we identified two *panC* group III B. cereus sensu lato strains that did not possess anthrax toxin- or capsule-encoding genes and did not belong to the classic “clonal” B. anthracis lineage associated with anthrax disease (34, 55). These strains would be classified as “B. cereus” or “group III B. cereus” using standard microbiological assays (53, 54); however, these strains were assigned to the “B. anthracis” genomospecies using GTDB and similar ANI-based methods (Table 1). While it is possible for B. anthracis strains to lose virulence plasmids during storage (56), the isolates sequenced here were not members of any anthrax toxin gene-harboring lineages (Fig. 5). Thus, referring to these strains as “B. anthracis” would be misleading as they cannot cause anthrax, and this potential miscommunication could have disastrous public health and industrial consequences.

We additionally isolated three B. cereus sensu lato strains from beef and poultry products, which were assigned to the “*B. paranthracis*” genomospecies via GTDB and similar ANI-based methods (Table 1). As noted previously, “*B. paranthracis*” was proposed as a “novel” species in 2017 (57) but was later found to encompass the well-known foodborne pathogen “emetic B. cereus” within its genomospecies boundary (29, 37, 38). One of the “*B. paranthracis*” strains isolated here indeed possessed cereulide synthetase-encoding genes and belonged to ST26 (Table 2), the ST that encompasses most B. cereus sensu lato strains capable of producing emetic toxin (38). This strain thus likely poses a food safety threat and would most likely be referred to as “emetic B. cereus” in clinical or industrial settings. Referring to this strain as “*B. paranthracis*” could be misleading to researchers, clinicians, and other professionals who are not well versed and up to date in B. cereus sensu lato taxonomy (29, 34, 38). However, not all “*B. paranthracis*” strains are capable of producing emetic toxin. Here, two ST2413 strains isolated from poultry were assigned to the “*B. paranthracis*” genomospecies but did not possess cereulide synthetase-encoding genes (Table 2), indicating that these strains cannot cause emetic intoxication. Thus, differentiating potentially emetic from nonemetic strains is critical for informing public health and food safety efforts.

Recently, we proposed a standardized nomenclatural framework for B. cereus sensu lato (i.e., the 2020 GSB framework), which can use genomic, genetic, and/or phenotypic information for taxonomic classification (33, 34). Importantly, the 2020 GSB framework relies on a standardized collection of biovars (i.e., biovars Anthracis, Emeticus, and Thuringiensis), which can be applied to individual B. cereus sensu lato strains to convey phenotypes of clinical and/or industrial importance (i.e., ability to produce anthrax, emetic, and insecticidal toxins, respectively) (33, 34). Within this framework, the absence of the Anthracis biovar term denotes that B. cereus sensu lato strains sequenced here cannot produce anthrax toxin, while the presence/absence of the Emeticus biovar term differentiates cereulide-producing “*B. paranthracis*” from noncereulide-producing strains (Table 2). While the 2020 GSB framework provides a standardized set of B. cereus sensu lato genomospecies (Tables 1 and 2) (33, 34), researchers and other professionals may prefer to use more established names for lineages (e.g., obtained via MLST, *panC* group assignment); biovar terms can thus be appended to B. cereus sensu lato lineage names (e.g., the ST26 strain sequenced here, which possesses cereulide synthetase, can be referred to as “B. cereus sensu lato ST26 biovar Emeticus”). Overall, standardized taxonomic frameworks that can incorporate both genomic/genetic and phenotypic information may improve strain-level risk evaluation of B. cereus sensu lato.

### WGS may improve B. cereus sensu lato surveillance, traceback investigations, and source tracking in the future.

Here, we showed that WGS may conceptually be used for B. cereus sensu lato surveillance and source tracking; however, our study has numerous limitations. First and foremost, we are limited by the relatively small number of isolates that we were able to sequence in this study. While WGS is being increasingly used for foodborne pathogen surveillance in Africa (58), the vast majority of WGS-based foodborne pathogen surveillance efforts are concentrated in world regions with lower burdens of foodborne illness (e.g., the United States and Europe) (18, 58). Thus, future WGS efforts are needed to gain further insight into the B. cereus sensu lato lineages circulating within the South African food supply chain.

Second, our study is limited by a lack of publicly available (i) genomic data and (ii) corresponding metadata associated with B. cereus sensu lato strains. For example, we identified two identical B. cereus sensu lato ST2413 strains, which were present in both Dutch and South African raw poultry. However, due to a lack of additional publicly available ST2413 B. cereus sensu lato genomes, we were unable to gain additional insights into exactly where this lineage originated. WGS has been shown to improve surveillance and source tracking efforts for numerous foodborne pathogens, including Escherichia coli, Salmonella enterica, and Listeria monocytogenes (50, 59). While the amount of publicly available WGS data derived from members of B. cereus sensu lato is increasing (34), efforts to sequence the genomes of food-associated B. cereus sensu lato strains are lagging relative to other foodborne pathogens. Only 2,664 assembled genomes from all B. cereus sensu lato species that met the quality thresholds used in this study were available in NCBI’s RefSeq Assembly database (60, 61) during the time this study was conducted (note that the NCBI GenBank Assembly database did not have any large multi-isolate B. cereus sensu lato projects at this time; accessed 20 March 2021). This can be contrasted with other foodborne pathogens for which source tracking and surveillance have proven to be successful (50, 59), as the numbers of publicly available genomes from these organisms are orders of magnitude greater than the number of genomes from all B. cereus sensu lato species combined (e.g., Salmonella enterica and Listeria monocytogenes, each single species with more than 100 and 10 times as many publicly available genomes, respectively; NCBI GenBank Assembly database, accessed 25 February 2022). Thus, future B. cereus sensu lato surveillance and WGS initiatives in clinical, industrial, and environmental settings are needed to improve B. cereus sensu lato source tracking and traceback efforts. Furthermore, it is essential that data and metadata obtained in such future initiatives are made publicly available, as international sharing of WGS data can decrease both the amount of time required to solve foodborne outbreaks and the public health burden caused by foodborne pathogens (62).

Stemming from the lack of B. cereus sensu lato WGS data and metadata, a final limitation of our study is that there are very few existing studies that have used WGS to characterize B. cereus sensu lato isolates known to come from a single source (e.g., from point-source outbreaks and clusters). Consequently, metrics that are used to determine whether two B. cereus sensu lato genomes are “identical” or derived from a common source (e.g., pairwise SNP cutoffs, core genome MLST allelic differences, and whole-genome phylogenetic topology) (63, 64) are sparse and only available for select lineages (e.g., emetic ST26, B. anthracis) (37, 38, 65). Therefore, future B. cereus sensu lato source tracking and surveillance efforts will benefit greatly not only from more extensive WGS efforts but also improved epidemiological surveillance of illness caused by members of B. cereus sensu lato (29), as more data linking strains to illness are needed.

Overall, the proof-of-concept study detailed here highlights the benefits of WGS for B. cereus sensu lato surveillance and source tracking, even among closely related lineages, and future studies will benefit from increasingly available publicly available WGS data and metadata.

## MATERIALS AND METHODS

### Isolate selection and whole-genome sequencing.

A subset of 34 isolates were selected from a total of 79 B. cereus sensu lato isolates from our previous study (26) using simple random sampling without replacement (66) via random numbers generated in Microsoft Excel. Culturing and genomic DNA extraction was performed as described previously (26) using the High Pure PCR template preparation kit (Roche, Germany). WGS of selected isolates was performed at the Biotechnology Platform, Agricultural Research Council, Onderstepoort, South Africa. DNA libraries were prepared using TruSeq and Nextera DNA library preparation kits (Illumina, San Diego, CA, USA), followed by sequencing on HiSeq and MiSeq instruments (Illumina, San Diego, CA, USA).

### Data preprocessing and quality control.

Paired-end reads associated with each of the 34 isolates were supplied as input to Trimmomatic v0.38 (67), which was used to remove Illumina adapters and leading and trailing low-quality/ambiguous bases (LEADING:3 and TRAILING:3, respectively); reads with average per base quality scores of <15 within a 4-bp sliding window (SLIDINGWINDOW:4:15) were additionally cut, and reads with lengths of <36 bp were removed. FastQC v0.11.5 (https://www.bioinformatics.babraham.ac.uk/projects/fastqc/) was used to assess the quality of the resulting trimmed paired-end reads.

SKESA v2.4.0 (68) was used to assemble each genome using the trimmed paired-end reads as input and default settings. SPAdes v3.13.1 (69) was additionally used to assemble each genome in “careful” mode using trimmed paired-end reads as input. QUAST v4.5 (70) was used to assess the quality of each resulting assembly, and CheckM v1.1.3 (71) was used to evaluate genome contamination/completeness. MultiQC v1.8 (72) was used to assess genome quality in aggregate. Assemblies produced using SKESA were used in all subsequent steps, as they were of higher quality based on metrics produced by QUAST (e.g., *N*_50_, number of contigs). Genomes with (i) <95% completeness (via CheckM), (ii) >5% contamination (via CheckM), and/or (iii) an *N*_50_ of <20 kbp were considered to be of low quality and were excluded (*n *= 4), yielding a preliminary set of 30 genomes used in subsequent analyses.

### *In silico* typing and taxonomic characterization.

BTyper3 v3.1.1 (33) was used to characterize each assembled genome (see Data preprocessing and quality control) using (i) ANI-based genomospecies, (ii) ANI-based subspecies, and (iii) biovar assignment using a standardized nomenclatural framework for B. cereus sensu lato (34) and dependencies FastANI v1.31 (55) and BLAST v2.9.0 (73), (iv) ANI-based pseudo-gene flow unit assignment (33) (also via FastANI), (v) *in silico* seven-gene MLST using the PubMLST B. cereus database (accessed 25 October 2020), and (vi) *panC* phylogenetic group assignment using an adjusted eight-group (groups I to VIII) framework (33). All aforementioned analyses were performed using default settings as well as with virulence factor minimum coverage thresholds lowered to 0% (–virulence_coverage 0) to confirm virulence factor absence. Because BTyper3 uses a conservative approach for Bt toxin gene detection, the command-line implementation of BtToxin_scanner v1.0 (BtToxin_scanner2.pl) was used to identify Bt toxin genes in each genome using default settings (74).

Taxonomic classification of assembled genomes was additionally performed using GTDB Release 05-RS95 (17 July 2020) and GTDB-Tk v. 1.3.0 (i.e., “GTDB R95”) using GTDB-Tk’s “classify_wf” workflow (75–77). Notably, five genomes were assigned to species outside B. cereus sensu lato (i.e., three genomes classified as Escherichia *flexneri*, one as Escherichia *dysenteriae*, and one as Staphylococcus saprophyticus via GTDB-Tk) and were thus excluded, yielding a final set of 25 B. cereus sensu lato genomes used in subsequent analyses (Table 1 and Table S1 in the supplemental material).

### Phylogenomic comparison of South African B. cereus sensu lato genomes to B. cereus sensu lato species type strains.

Prokka v1.14.6 (78) was used to annotate each of the 25 B. cereus sensu lato genomes sequenced in this study. Protein-coding sequences derived from the type strain genomes of each of the 23 validly published and effective B. cereus sensu lato species (accessed 28 August 2021) were downloaded from NCBI’s RefSeq Assembly database (see Table 1 of Méndez Acevedo et al. for all type strain accession numbers) (79). OrthoFinder v2.5.2 (80, 81) was used to identify orthologues among protein-coding sequences associated with all 47 genomes (25 B. cereus sensu lato genomes sequenced in this study plus 23 B. cereus sensu lato species type strain genomes) using MAFFT v7.475 (82, 83) for sequence alignment and RAxML-NG v1.0.2 (84) for phylogeny construction.

The resulting amino acid sequence alignment was supplied as input to IQ-TREE v1.5.4 (85), which was used to construct a maximum likelihood (ML) phylogeny, using 1,000 replicates of the ultrafast bootstrap approximation (86) plus the optimal amino acid substitution model selected using ModelFinder (i.e., the general matrix model with empirical amino acid frequencies and the FreeRate model with six categories; JTT+F+R6) (87–90). The resulting phylogeny was rooted using effective species “*B. manliponensis*” (i.e., the most distant recognized member of B. cereus sensu lato) (91) and annotated using the bactaxR package (34) in R v4.1.2 (92).

### Acquisition of publicly available B. cereus sensu lato genomes.

All assembled genomes submitted to the National Center for Biotechnology Information RefSeq database (60, 61) as one of 23 validly published or effective B. cereus sensu lato species (i.e., *albus*, *anthracis*, “*bingmayongensis*,” *cereus*, “*clarus*,” *cytotoxicus*, *fungorum*, “*gaemokensis*,” *luti*, “*manliponensis*,” *mobilis*, *mycoides*, *nitratireducens*, *pacificus*, *paramycoides*, *paranthracis*, *proteolyticus*, *pseudomycoides*, *thuringiensis*, *toyonensis*, *tropicus*, *weihenstephanensis*, and *wiedmannii*) (1, 29, 57, 79, 91, 93–105) were downloaded (*n *= 2,733; accessed 20 March 2021). QUAST and CheckM were used to assess the quality of each assembled genome (see Data preprocessing and quality control), and BTyper3 (using default settings) and GTDB-Tk were used for typing and/or taxonomic assignment as described above (see *In silico* typing and taxonomic characterization). The rentrez package (v1.2.3) was used to download metadata associated with each genome’s BioSample in R v3.6.1 (92, 106, 107). Publicly available genomes meeting all of the following quality thresholds were used in subsequent analyses (*n *= 2,664; Table S2): (i) >95% completeness (via CheckM), (ii) <5% contamination (via CheckM), (iii) *N*_50_ of >20 kbp (via QUAST), and (iv) composed of <1,000 contigs (via QUAST).

### Acquisition of genomes from a study of B. thuringiensis outbreaks.

All sequencing reads associated with isolates from a previous study of outbreaks caused by B. thuringiensis in France (108) were downloaded from NCBI’s Sequence Read Archive (SRA) database using the SRA Toolkit v 2.8.2 (109, 110). Genomic data for all 171 isolates were preprocessed, assembled, and taxonomically classified as described above, with genomes assembled using SKESA used in subsequent steps (see Data preprocessing and quality control and *In silico* typing and taxonomic characterization). Four genomes did not meet the quality thresholds used in this study (see Acquisition of publicly available B. cereus sensu lato genomes) and were thus excluded, yielding 167 genomes from the study that were used in subsequent analyses (Table S3).

### Acquisition of genomes derived from strains isolated in conjunction with a previous outbreak caused by emetic B. cereus sensu lato.

The genomes of 33 B. cereus sensu lato strains isolated in conjunction with a 2016 emetic outbreak in New York State (United States) were downloaded, preprocessed, and assembled as described previously (37). The quality of each of the 33 genomes was assessed as described above (see Data preprocessing and quality control), and all genomes underwent taxonomic classification and typing as described above (see *In silico* typing and taxonomic characterization). Two genomes did not meet the quality thresholds used in this study (see Acquisition of publicly available B. cereus sensu lato genomes) and were thus excluded, yielding 31 genomes from the study that were used in subsequent analyses (Table S4).

### Within-group phylogeny construction.

The 25 B. cereus sensu lato strains sequenced here spanned four major phylogenetic groups based on their *panC* sequence (i.e., *panC* groups II, III, IV, and V; Table 1). Thus, phylogenies were constructed using all genomes assigned to each of the following major lineages: (i) *panC* group IV (Fig. 3), (ii) *panC* groups II and III, excluding *B. luti* (Fig. 5), and (iii) *panC* group V (Fig. 6), which are equivalent to the (i) B. cereus sensu stricto, (ii) *B. mosaicus*, and (iii) *B. toyonensis* genomospecies within the 2020 GSB taxonomic framework (33), respectively (*panC* group II and III genomes were aggregated due to the fact that these lineages are closely related and polyphyletic; Fig. 5).

For each of the three major lineages, Prokka was used to annotate each genome; the resulting general feature format (GFF) files associated with each genome were supplied as input to Panaroo v1.2.8 (111), which was used to partition genes into core- and pan-genome orthologous gene clusters using the following parameters (all other parameters were set to their default values): (i) “strict” mode (–clean-mode strict), (ii) core genome alignment using MAFFT (-a core –aligner mafft), and (iii) a core genome sample threshold of 95% (–core_threshold 0.95). The resulting core genome (nucleotide) alignment was queried using snp-sites v2.5.1 (112), which was used to identify (i) core SNPs and (ii) constant sites among all genomes in the major lineage. The resulting core SNP alignment was supplied as input to IQ-TREE v1.5.4, which was used to construct an ML phylogeny using the general time-reversible (GTR) nucleotide substitution model (113), 1,000 replicates of the ultrafast bootstrap approximation (86), and an ascertainment bias correction obtained using constant sites output by snp-sites.

For each of the three major lineages, all aforementioned steps were repeated, with the addition of an outgroup genome. For the *panC* group IV phylogeny, *panC* group III B. anthracis strain Ames Ancestor was used as an outgroup (NCBI RefSeq accession number GCF_000008445.1). For the *panC* groups II/III and *panC* group V phylogenies, *panC* group IV B. cereus strain ATCC 14579 was used as an outgroup (NCBI RefSeq accession number GCF_006094295.1). Additionally, only genomes with detailed metadata (i.e., a reported year of isolation, isolation source, and geographic location) were included in this analysis (Tables S1 to S4). The resulting phylogenies were annotated using the bactaxR package in R.

### Delineation of MLST lineages and identification of closely related and “identical” genomes.

FastANI v1.31 was used to calculate ANI values between each of the 25 B. cereus sensu lato genomes sequenced in this study (i.e., as a query genome), and all genomes assigned to the *panC* group of the query genome (*panC* groups II and III were aggregated); genomes were then grouped into lineages based on STs assigned using seven-gene MLST (see *In silico* typing and taxonomic characterization). For each of the resulting MLST lineages, FastANI was used to calculate pairwise ANI values between all genomes within the MLST lineage (Table 3).

For each MLST lineage, Snippy v4.6.0 (https://github.com/tseemann/snippy) was used to identify core SNPs among all genomes assigned to the respective MLST lineage using (i) a genome sequenced in this study as a reference genome (Table 3), (ii) paired-end reads associated with each genome as input (for the genomes sequenced in this study as well as the genomes from the Bonis et al. study and the New York State outbreak study) (37, 108) and/or assembled genomes as input (for NCBI genomes), and (iii) default settings. For MLST lineages with more than four genomes (i.e., ST24, ST26, ST177, ST223, and ST1578), Gubbins v3.1.3 (114) was used to remove recombination, and core SNPs were identified within the resulting filtered alignment using snp-sites. For all MLST lineages, pairwise core SNP distances were calculated within the MLST lineage (i) among all genomes, (ii) among genomes sequenced in this study, and (iii) between genomes sequenced in this study and publicly available genomes (Table 3) using the dist.gene function in the ape package (115, 116) in R.

### Data availability.

Paired-end Illumina reads associated with the 25 B. cereus sensu lato isolates sequenced in this study have been deposited in NCBI’s SRA database under BioProject accession number PRJNA798224. Metadata and quality information for all genomes queried in this study are available in Table S1 (the 25 isolates sequenced in this study) and Tables S2 to S4 (all publicly available genomes).

## References

[1] Stenfors Arnesen LP, Fagerlund A, Granum PE. 2008. From soil to gut: *Bacillus cereus* and its food poisoning toxins. FEMS Microbiol Rev 32:579–606. doi:10.1111/j.1574-6976.2008.00112.x. 18422617

[2] Jouzani GS, Valijanian E, Sharafi R. 2017. *Bacillus thuringiensis*: a successful insecticide with new environmental features and tidings. Appl Microbiol Biotechnol 101:2691–2711. doi:10.1007/s00253-017-8175-y. 28235989

[3] Raymond B, Federici BA. 2017. In defence of *Bacillus thuringiensis*, the safest and most successful microbial insecticide available to humanity—a response to EFSA. FEMS Microbiol Ecol 93:fix084. doi:10.1093/femsec/fix084. 28645183 PMC5812528

[4] Azizoglu U. 2019. *Bacillus thuringiensis* as a biofertilizer and biostimulator: a mini-review of the little-known plant growth-promoting properties of Bt. Curr Microbiol 76:1379–1385. doi:10.1007/s00284-019-01705-9. 31101973

[5] Elshaghabee FMF, Rokana N, Gulhane RD, Sharma C, Panwar H. 2017. *Bacillus* as potential probiotics: status, concerns, and future perspectives. Front Microbiol 8:1490. doi:10.3389/fmicb.2017.01490. 28848511 PMC5554123

[6] Kantas D, Papatsiros VG, Tassis PD, Giavasis I, Bouki P, Tzika ED. 2015. A feed additive containing *Bacillus toyonensis* (Toyocerin) protects against enteric pathogens in postweaning piglets. J Appl Microbiol 118:727–738. doi:10.1111/jam.12729. 25512110

[7] Jovanovic J, Ornelis VFM, Madder A, Rajkovic A. 2021. *Bacillus cereus* food intoxication and toxicoinfection. Compr Rev Food Sci Food Saf 20:3719–3761. doi:10.1111/1541-4337.12785. 34160120

[8] Baldwin VM. 2020. You can’t *B. cereus—*a review of *Bacillus cereus* strains that cause anthrax-like disease. Front Microbiol 11:1731. doi:10.3389/fmicb.2020.01731. 32973690 PMC7468541

[9] Ehling-Schulz M, Lereclus D, Koehler TM. 2019. The *Bacillus cereus* group: *Bacillus* species with pathogenic potential. Microbiol Spectr 7. doi:10.1128/microbiolspec.GPP3-0032-2018. PMC6530592 31111815

[10] Moayeri M, Leppla SH, Vrentas C, Pomerantsev AP, Liu S. 2015. Anthrax pathogenesis. Annu Rev Microbiol 69:185–208. doi:10.1146/annurev-micro-091014-104523. 26195305

[11] Pilo P, Frey J. 2018. Pathogenicity, population genetics and dissemination of *Bacillus anthracis*. Infect Genet Evol 64:115–125. doi:10.1016/j.meegid.2018.06.024. 29935338

[12] Ehling-Schulz M, Frenzel E, Gohar M. 2015. Food-bacteria interplay: pathometabolism of emetic *Bacillus cereus*. Front Microbiol 6:704. doi:10.3389/fmicb.2015.00704. 26236290 PMC4500953

[13] Rouzeau-Szynalski K, Stollewerk K, Messelhausser U, Ehling-Schulz M. 2020. Why be serious about emetic *Bacillus cereus*: cereulide production and industrial challenges. Food Microbiol 85:103279. doi:10.1016/j.fm.2019.103279. 31500702

[14] Dietrich R, Jessberger N, Ehling-Schulz M, Martlbauer E, Granum PE. 2021. The food poisoning toxins of *Bacillus cereus*. Toxins 13:98. doi:10.3390/toxins13020098. 33525722 PMC7911051

[15] Jessberger N, Dietrich R, Granum PE, Martlbauer E. 2020. The *Bacillus cereus* food infection as multifactorial process. Toxins 12:701. doi:10.3390/toxins12110701. 33167492 PMC7694497

[16] Bottone EJ. 2010. *Bacillus cereus*, a volatile human pathogen. Clin Microbiol Rev 23:382–398. doi:10.1128/CMR.00073-09. 20375358 PMC2863360

[17] Glasset B, Herbin S, Granier SA, Cavalie L, Lafeuille E, Guerin C, Ruimy R, Casagrande-Magne F, Levast M, Chautemps N, Decousser JW, Belotti L, Pelloux I, Robert J, Brisabois A, Ramarao N. 2018. *Bacillus cereus,* a serious cause of nosocomial infections: epidemiologic and genetic survey. PLoS One 13:e0194346. doi:10.1371/journal.pone.0194346. 29791442 PMC5966241

[18] Kirk MD, Pires SM, Black RE, Caipo M, Crump JA, Devleesschauwer B, Dopfer D, Fazil A, Fischer-Walker CL, Hald T, Hall AJ, Keddy KH, Lake RJ, Lanata CF, Torgerson PR, Havelaar AH, Angulo FJ. 2015. World Health Organization estimates of the global and regional disease burden of 22 foodborne bacterial, protozoal, and viral diseases, 2010: a data synthesis. PLoS Med 12:e1001921. doi:10.1371/journal.pmed.1001921. 26633831 PMC4668831

[19] Konuma H, Shinagawa K, Tokumaru M, Onoue Y, Konno S, Fujino N, Shigehisa T, Kurata H, Kuwabara Y, Lopes CAM. 1988. Occurrence of *Bacillus cereus* in meat products, raw meat and meat product additives. J Food Prot 51:324–326. doi:10.4315/0362-028X-51.4.324. 30978861

[20] Tewari A, Singh SP, Singh R. 2015. Incidence and enterotoxigenic profile of *Bacillus cereus i*n meat and meat products of Uttarakhand, India. J Food Sci Technol 52:1796–1801. doi:10.1007/s13197-013-1162-0. 25745259 PMC4348265

[21] Kong L, Yu S, Yuan X, Li C, Yu P, Wang J, Guo H, Wu S, Ye Q, Lei T, Yang X, Zhang Y, Wei X, Zeng H, Zhang J, Wu Q, Ding Y. 2021. An investigation on the occurrence and molecular characterization of *Bacillus cereus* in meat and meat products in China. Foodborne Pathog Dis 18:306–314. doi:10.1089/fpd.2020.2885. 33769083

[22] Smith DP, Berrang ME, Feldner PW, Phillips RW, Meinersmann RJ. 2004. Detection of *Bacillus cereus* on selected retail chicken products. J Food Prot 67:1770–1773. doi:10.4315/0362-028x-67.8.1770. 15330548

[23] Osman KM, Kappell AD, Orabi A, Al-Maary KS, Mubarak AS, Dawoud TM, Hemeg HA, Moussa IMI, Hessain AM, Yousef HMY, Hristova KR. 2018. Poultry and beef meat as potential seedbeds for antimicrobial resistant enterotoxigenic *Bacillus species*: a materializing epidemiological and potential severe health hazard. Sci Rep 8:11600. doi:10.1038/s41598-018-29932-3. 30072706 PMC6072766

[24] Zeighami H, Nejad-Dost G, Parsadanians A, Daneshamouz S, Haghi F. 2020. Frequency of hemolysin BL and non-hemolytic enterotoxin complex genes of *Bacillus cereus* in raw and cooked meat samples in Zanjan, Iran. Toxicol Rep 7:89–92. doi:10.1016/j.toxrep.2019.12.006. 31908970 PMC6938900

[25] Yu S, Yu P, Wang J, Li C, Guo H, Liu C, Kong L, Yu L, Wu S, Lei T, Chen M, Zeng H, Pang R, Zhang Y, Wei X, Zhang J, Wu Q, Ding Y. 2020. A study on prevalence and characterization of *Bacillus cereus* in ready-to-eat foods in China. Front Microbiol 10:3043. doi:10.3389/fmicb.2019.03043. 32010099 PMC6974471

[26] Madoroba E, Magwedere K, Chaora NS, Matle I, Muchadeyi F, Mathole MA, Pierneef R. 2021. Microbial communities of meat and meat products: an exploratory analysis of the product quality and safety at selected enterprises in South Africa. Microorganisms 9:507. doi:10.3390/microorganisms9030507. 33673660 PMC7997435

[27] Nortjé GL, Vorster SM, Greebe RP, Steyn PL. 1999. Occurrence of *Bacillus cereus* and *Yersinia enterocolitica* in South African retail meats. Food Microbiol 16:213–217. doi:10.1006/fmic.1998.0240.

[28] Shale K, Malebo NJ. 2011. Quantification and antibiotic susceptibility profiles of *S. aureus* and *B. cereus* strains isolated from biltong. J Food Safety 31:559–569. doi:10.1111/j.1745-4565.2011.00335.x.

[29] Carroll LM, Cheng RA, Wiedmann M, Kovac J. 2021. Keeping up with the *Bacillus cereus* group: taxonomy through the genomics era and beyond. Crit Rev Food Sci Nutr doi:10.1080/10408398.2021.1916735. 33939559

[30] Liu Y, Lai Q, Goker M, Meier-Kolthoff JP, Wang M, Sun Y, Wang L, Shao Z. 2015. Genomic insights into the taxonomic status of the *Bacillus cereus* group. Sci Rep 5:14082. doi:10.1038/srep14082. 26373441 PMC4571650

[31] Guinebretiere MH, Thompson FL, Sorokin A, Normand P, Dawyndt P, Ehling-Schulz M, Svensson B, Sanchis V, Nguyen-The C, Heyndrickx M, De Vos P. 2008. Ecological diversification in the *Bacillus cereus* group. Environ Microbiol 10:851–865. doi:10.1111/j.1462-2920.2007.01495.x. 18036180

[32] Guinebretiere MH, Velge P, Couvert O, Carlin F, Debuyser ML, Nguyen-The C. 2010. Ability of *Bacillus cereus* group strains to cause food poisoning varies according to phylogenetic affiliation (groups I to VII) rather than species affiliation. J Clin Microbiol 48:3388–3391. doi:10.1128/JCM.00921-10. 20660215 PMC2937725

[33] Carroll LM, Cheng RA, Kovac J. 2020. No assembly required: using BTyper3 to assess the congruency of a proposed taxonomic framework for the *Bacillus cereus* group with historical typing methods. Front Microbiol 11:580691. doi:10.3389/fmicb.2020.580691. 33072050 PMC7536271

[34] Carroll LM, Wiedmann M, Kovac J. 2020. Proposal of a taxonomic nomenclature for the *Bacillus cereus* group which reconciles genomic definitions of bacterial species with clinical and industrial phenotypes. mBio 11:e00034-20. doi:10.1128/mBio.00034-20. 32098810 PMC7042689

[35] Jolley KA, Maiden MC. 2010. BIGSdb: scalable analysis of bacterial genome variation at the population level. BMC Bioinformatics 11:595. doi:10.1186/1471-2105-11-595. 21143983 PMC3004885

[36] Jolley KA, Bray JE, Maiden MCJ. 2018. Open-access bacterial population genomics: BIGSdb software, the PubMLST.org website and their applications. Wellcome Open Res 3:124. doi:10.12688/wellcomeopenres.14826.1. 30345391 PMC6192448

[37] Carroll LM, Wiedmann M, Mukherjee M, Nicholas DC, Mingle LA, Dumas NB, Cole JA, Kovac J. 2019. Characterization of emetic and diarrheal *Bacillus cereus* strains from a 2016 foodborne outbreak using whole-genome sequencing: addressing the microbiological, epidemiological, and bioinformatic challenges. Front Microbiol 10:144. doi:10.3389/fmicb.2019.00144. 30809204 PMC6379260

[38] Carroll LM, Wiedmann M. 2020. Cereulide synthetase acquisition and loss events within the evolutionary history of group III *Bacillus cereus sensu lato* facilitate the transition between emetic and diarrheal foodborne pathogens. mBio 11:e01263-20. doi:10.1128/mBio.01263-20. 32843545 PMC7448271

[39] Bucher T, Keren-Paz A, Hausser J, Olender T, Cytryn E, Kolodkin-Gal I. 2019. An active beta-lactamase is a part of an orchestrated cell wall stress resistance network of *Bacillus subtilis* and related rhizosphere species. Environ Microbiol 21:1068–1085. doi:10.1111/1462-2920.14526. 30637927

[40] Ehling-Schulz M, Svensson B, Guinebretiere MH, Lindback T, Andersson M, Schulz A, Fricker M, Christiansson A, Granum PE, Martlbauer E, Nguyen-The C, Salkinoja-Salonen M, Scherer S. 2005. Emetic toxin formation of *Bacillus cereus* is restricted to a single evolutionary lineage of closely related strains. Microbiology (Reading) 151:183–197. doi:10.1099/mic.0.27607-0. 15632437

[41] Glasset B, Herbin S, Guillier L, Cadel-Six S, Vignaud ML, Grout J, Pairaud S, Michel V, Hennekinne JA, Ramarao N, Brisabois A. 2016. *Bacillus cereus*-induced food-borne outbreaks in France, 2007 to 2014: epidemiology and genetic characterisation. Euro Surveill 21:30413. doi:10.2807/1560-7917.ES.2016.21.48.30413. 27934583 PMC5388111

[42] Guinebretiere MH, Loux V, Martin V, Nicolas P, Sanchis V, Broussolle V. 2017. Draft genome sequences of 18 psychrotolerant and 2 thermotolerant strains representative of particular ecotypes in the *Bacillus cereus* group. Genome Announc 5:e01568-16. doi:10.1128/genomeA.01568-16. 28153905 PMC5289691

[43] Grubbs KJ, Bleich RM, Santa Maria KC, Allen SE, Farag S, AgBiome T, Shank EA, Bowers AA, AgBiome Team. 2017. Large-scale bioinformatics analysis of *Bacillus* genomes uncovers conserved roles of natural products in bacterial physiology. mSystems 2:e00040-17. doi:10.1128/mSystems.00040-17. 29152584 PMC5686519

[44] Stein RA, Chirilã M. 2017. Chapter 3—Routes of transmission in the food chain, p 65–103. *In* Dodd CER, Aldsworth T, Stein RA, Cliver DO, Riemann HP (ed), Foodborne diseases, 3rd ed. Academic Press, Cambridge, MA.

[45] Ahn J-W, Rhodes MT. 2021. Examining pathogen-based import refusals: trends and analysis from 2002 to 2019. U.S. Department of Agriculture, Economic Research Service, U.S. Department of Agriculture ERS, Washington, DC.

[46] Rohr JR, Barrett CB, Civitello DJ, Craft ME, Delius B, DeLeo GA, Hudson PJ, Jouanard N, Nguyen KH, Ostfeld RS, Remais JV, Riveau G, Sokolow SH, Tilman D. 2019. Emerging human infectious diseases and the links to global food production. Nat Sustain 2:445–456. doi:10.1038/s41893-019-0293-3. 32219187 PMC7091874

[47] Ristaino JB, Anderson PK, Bebber DP, Brauman KA, Cunniffe NJ, Fedoroff NV, Finegold C, Garrett KA, Gilligan CA, Jones CM, Martin MD, MacDonald GK, Neenan P, Records A, Schmale DG, Tateosian L, Wei Q. 2021. The persistent threat of emerging plant disease pandemics to global food security. Proc Natl Acad Sci USA 118:e2022239118. doi:10.1073/pnas.2022239118. 34021073 PMC8201941

[48] Ercsey-Ravasz M, Toroczkai Z, Lakner Z, Baranyi J. 2012. Complexity of the international agro-food trade network and its impact on food safety. PLoS One 7:e37810. doi:10.1371/journal.pone.0037810. 22701535 PMC3365103

[49] Brown E, Dessai U, McGarry S, Gerner-Smidt P. 2019. Use of whole-genome sequencing for food safety and public health in the United States. Foodborne Pathog Dis 16:441–450. doi:10.1089/fpd.2019.2662. 31194586 PMC6653787

[50] Brown B, Allard M, Bazaco MC, Blankenship J, Minor T. 2021. An economic evaluation of the whole genome sequencing source tracking program in the U.S. PLoS One 16:e0258262. doi:10.1371/journal.pone.0258262. 34614029 PMC8494326

[51] Makgopa M. 2020. South Africa lifts ban on poultry imports from the Netherlands. United States Department of Agriculture (USDA) Foreign Agricultural Service, Washington, DC.

[52] Kabir MS, Hsieh YH, Simpson S, Kerdahi K, Sulaiman IM. 2017. Evaluation of two standard and two chromogenic selective media for optimal growth and enumeration of isolates of 16 unique *Bacillus* species. J Food Prot 80:952–962. doi:10.4315/0362-028X.JFP-16-441. 28467187

[53] Tallent SM, Knolhoff A, Rhodehamel EJ, Harmon SM, Bennett RW. 2019. *Bacillus cereus*, Chapter 14. *In* Bacteriological analytical manual (BAM), 8th ed. Food and Drug Administration, Washington, DC.

[54] Tallent SM, Kotewicz KM, Strain EA, Bennett RW. 2012. Efficient isolation and identification of *Bacillus cereus* group. J AOAC Int 95:446–451. doi:10.5740/jaoacint.11-251. 22649932

[55] Jain C, Rodriguez RL, Phillippy AM, Konstantinidis KT, Aluru S. 2018. High throughput ANI analysis of 90K prokaryotic genomes reveals clear species boundaries. Nat Commun 9:5114. doi:10.1038/s41467-018-07641-9. 30504855 PMC6269478

[56] Marston CK, Hoffmaster AR, Wilson KE, Bragg SL, Plikaytis B, Brachman P, Johnson S, Kaufmann AF, Popovic T. 2005. Effects of long-term storage on plasmid stability in *Bacillus anthracis*. Appl Environ Microbiol 71:7778–7780. doi:10.1128/AEM.71.12.7778-7780.2005. 16332750 PMC1317469

[57] Liu Y, Du J, Lai Q, Zeng R, Ye D, Xu J, Shao Z. 2017. Proposal of nine novel species of the *Bacillus cereus* group. Int J Syst Evol Microbiol 67:2499–2508. doi:10.1099/ijsem.0.001821. 28792367

[58] Inzaule SC, Tessema SK, Kebede Y, Ogwell Ouma AE, Nkengasong JN. 2021. Genomic-informed pathogen surveillance in Africa: opportunities and challenges. Lancet Infect Dis 21:e281–e289. doi:10.1016/S1473-3099(20)30939-7. 33587898 PMC7906676

[59] Rantsiou K, Kathariou S, Winkler A, Skandamis P, Saint-Cyr MJ, Rouzeau-Szynalski K, Amezquita A. 2018. Next generation microbiological risk assessment: opportunities of whole genome sequencing (WGS) for foodborne pathogen surveillance, source tracking and risk assessment. Int J Food Microbiol 287:3–9. doi:10.1016/j.ijfoodmicro.2017.11.007. 29246458

[60] NCBI Resource Coordinators. 2018. Database resources of the National Center for Biotechnology Information. Nucleic Acids Res 46:D8–D13. doi:10.1093/nar/gkx1095. 29140470 PMC5753372

[61] Pruitt KD, Tatusova T, Maglott DR. 2007. NCBI reference sequences (RefSeq): a curated non-redundant sequence database of genomes, transcripts and proteins. Nucleic Acids Res 35:D61–D65. doi:10.1093/nar/gkl842. 17130148 PMC1716718

[62] Pettengill JB, Markell A, Conrad A, Carleton HA, Beal J, Rand H, Musser S, Brown EW, Allard MW, Huffman J, Harris S, Wise M, Locas A. 2020. A multinational listeriosis outbreak and the importance of sharing genomic data. Lancet Microbe 1:e233–e234. doi:10.1016/S2666-5247(20)30122-1. 35544215 PMC10966468

[63] Stimson J, Gardy J, Mathema B, Crudu V, Cohen T, Colijn C. 2019. Beyond the SNP threshold: identifying outbreak clusters using inferred transmissions. Mol Biol Evol 36:587–603. doi:10.1093/molbev/msy242. 30690464 PMC6389316

[64] Olson ND, Lund SP, Colman RE, Foster JT, Sahl JW, Schupp JM, Keim P, Morrow JB, Salit ML, Zook JM. 2015. Best practices for evaluating single nucleotide variant calling methods for microbial genomics. Front Genet 6:235. doi:10.3389/fgene.2015.00235. 26217378 PMC4493402

[65] Abdel-Glil MY, Chiaverini A, Garofolo G, Fasanella A, Parisi A, Harmsen D, Jolley KA, Elschner MC, Tomaso H, Linde J, Galante D. 2021. A whole-genome-based gene-by-gene typing system for standardized high-resolution strain typing of *Bacillus anthracis*. J Clin Microbiol 59:e0288920. doi:10.1128/JCM.02889-20. 33827898 PMC8218748

[66] Rose S. 2015. Management research: applying the principles, 1st ed. Routledge, Taylor & Francis Group, New York, NY.

[67] Bolger AM, Lohse M, Usadel B. 2014. Trimmomatic: a flexible trimmer for Illumina sequence data. Bioinformatics 30:2114–2120. doi:10.1093/bioinformatics/btu170. 24695404 PMC4103590

[68] Souvorov A, Agarwala R, Lipman DJ. 2018. SKESA: strategic k-mer extension for scrupulous assemblies. Genome Biol 19:153. doi:10.1186/s13059-018-1540-z. 30286803 PMC6172800

[69] Bankevich A, Nurk S, Antipov D, Gurevich AA, Dvorkin M, Kulikov AS, Lesin VM, Nikolenko SI, Pham S, Prjibelski AD, Pyshkin AV, Sirotkin AV, Vyahhi N, Tesler G, Alekseyev MA, Pevzner PA. 2012. SPAdes: a new genome assembly algorithm and its applications to single-cell sequencing. J Comput Biol 19:455–477. doi:10.1089/cmb.2012.0021. 22506599 PMC3342519

[70] Gurevich A, Saveliev V, Vyahhi N, Tesler G. 2013. QUAST: quality assessment tool for genome assemblies. Bioinformatics 29:1072–1075. doi:10.1093/bioinformatics/btt086. 23422339 PMC3624806

[71] Parks DH, Imelfort M, Skennerton CT, Hugenholtz P, Tyson GW. 2015. CheckM: assessing the quality of microbial genomes recovered from isolates, single cells, and metagenomes. Genome Res 25:1043–1055. doi:10.1101/gr.186072.114. 25977477 PMC4484387

[72] Ewels P, Magnusson M, Lundin S, Kaller M. 2016. MultiQC: summarize analysis results for multiple tools and samples in a single report. Bioinformatics 32:3047–3048. doi:10.1093/bioinformatics/btw354. 27312411 PMC5039924

[73] Camacho C, Coulouris G, Avagyan V, Ma N, Papadopoulos J, Bealer K, Madden TL. 2009. BLAST+: architecture and applications. BMC Bioinformatics 10:421. doi:10.1186/1471-2105-10-421. 20003500 PMC2803857

[74] Ye W, Zhu L, Liu Y, Crickmore N, Peng D, Ruan L, Sun M. 2012. Mining new crystal protein genes from *Bacillus thuringiensis* on the basis of mixed plasmid-enriched genome sequencing and a computational pipeline. Appl Environ Microbiol 78:4795–4801. doi:10.1128/AEM.00340-12. 22544259 PMC3416374

[75] Chaumeil PA, Mussig AJ, Hugenholtz P, Parks DH. 2019. GTDB-Tk: a toolkit to classify genomes with the Genome Taxonomy Database. Bioinformatics 36:1925–1927. doi:10.1093/bioinformatics/btz848. 31730192 PMC7703759

[76] Parks DH, Chuvochina M, Waite DW, Rinke C, Skarshewski A, Chaumeil PA, Hugenholtz P. 2018. A standardized bacterial taxonomy based on genome phylogeny substantially revises the tree of life. Nat Biotechnol 36:996–1004. doi:10.1038/nbt.4229. 30148503

[77] Parks DH, Chuvochina M, Chaumeil PA, Rinke C, Mussig AJ, Hugenholtz P. 2020. A complete domain-to-species taxonomy for Bacteria and Archaea. Nat Biotechnol 38:1079–1086. doi:10.1038/s41587-020-0501-8. 32341564

[78] Seemann T. 2014. Prokka: rapid prokaryotic genome annotation. Bioinformatics 30:2068–2069. doi:10.1093/bioinformatics/btu153. 24642063

[79] Mendez Acevedo M, Carroll LM, Mukherjee M, Mills E, Xiaoli L, Dudley EG, Kovac J. 2020. Novel effective *Bacillus cereus* group species “*Bacillus clarus*” is represented by antibiotic-producing strain ATCC 21929 isolated from soil. mSphere 5:e00882-20. doi:10.1128/mSphere.00882-20. 33148822 PMC7643830

[80] Emms DM, Kelly S. 2015. OrthoFinder: solving fundamental biases in whole genome comparisons dramatically improves orthogroup inference accuracy. Genome Biol 16:157. doi:10.1186/s13059-015-0721-2. 26243257 PMC4531804

[81] Emms DM, Kelly S. 2019. OrthoFinder: phylogenetic orthology inference for comparative genomics. Genome Biol 20:238. doi:10.1186/s13059-019-1832-y. 31727128 PMC6857279

[82] Katoh K, Misawa K, Kuma K, Miyata T. 2002. MAFFT: a novel method for rapid multiple sequence alignment based on fast Fourier transform. Nucleic Acids Res 30:3059–3066. doi:10.1093/nar/gkf436. 12136088 PMC135756

[83] Katoh K, Standley DM. 2013. MAFFT multiple sequence alignment software version 7: improvements in performance and usability. Mol Biol Evol 30:772–780. doi:10.1093/molbev/mst010. 23329690 PMC3603318

[84] Kozlov AM, Darriba D, Flouri T, Morel B, Stamatakis A. 2019. RAxML-NG: a fast, scalable and user-friendly tool for maximum likelihood phylogenetic inference. Bioinformatics 35:4453–4455. doi:10.1093/bioinformatics/btz305. 31070718 PMC6821337

[85] Nguyen LT, Schmidt HA, von Haeseler A, Minh BQ. 2015. IQ-TREE: a fast and effective stochastic algorithm for estimating maximum-likelihood phylogenies. Mol Biol Evol 32:268–274. doi:10.1093/molbev/msu300. 25371430 PMC4271533

[86] Minh BQ, Nguyen MA, von Haeseler A. 2013. Ultrafast approximation for phylogenetic bootstrap. Mol Biol Evol 30:1188–1195. doi:10.1093/molbev/mst024. 23418397 PMC3670741

[87] Kalyaanamoorthy S, Minh BQ, Wong TKF, von Haeseler A, Jermiin LS. 2017. ModelFinder: fast model selection for accurate phylogenetic estimates. Nat Methods 14:587–589. doi:10.1038/nmeth.4285. 28481363 PMC5453245

[88] Jones DT, Taylor WR, Thornton JM. 1992. The rapid generation of mutation data matrices from protein sequences. Comput Appl Biosci 8:275–282. doi:10.1093/bioinformatics/8.3.275. 1633570

[89] Soubrier J, Steel M, Lee MSY, Der Sarkissian C, Guindon S, Ho SYW, Cooper A. 2012. The influence of rate heterogeneity among sites on the time dependence of molecular rates. Mol Biol Evol 29:3345–3358. doi:10.1093/molbev/mss140. 22617951

[90] Yang Z. 1995. A space-time process model for the evolution of DNA sequences. Genetics 139:993–1005. doi:10.1093/genetics/139.2.993. 7713447 PMC1206396

[91] Jung MY, Kim JS, Paek WK, Lim J, Lee H, Kim PI, Ma JY, Kim W, Chang YH. 2011. *Bacillus manliponensis* sp. nov., a new member of the *Bacillus cereus* group isolated from foreshore tidal flat sediment. J Microbiol 49:1027–1032. doi:10.1007/s12275-011-1049-6. 22203569

[92] R Core Team. 2019. R: a language and environment for statistical computing, v3.6.1. R Foundation for Statistical Computing, Vienna, Austria.

[93] Schoch CL, Ciufo S, Domrachev M, Hotton CL, Kannan S, Khovanskaya R, Leipe D, McVeigh R, O'Neill K, Robbertse B, Sharma S, Soussov V, Sullivan JP, Sun L, Turner S, Karsch-Mizrachi I. 2020. NCBI Taxonomy: a comprehensive update on curation, resources and tools. Database (Oxford) 2020:baaa062. doi:10.1093/database/baaa062. 32761142 PMC7408187

[94] Sternbach G. 2003. The history of anthrax. J Emerg Med 24:463–467. doi:10.1016/s0736-4679(03)00079-9. 12745053

[95] Frankland GC, Frankland PF, Lankester ER. 1887. XI. Studies on some new micro-organisms obtained from air. Philos Trans R Soc B: Biol Sci 178:257–287. doi:10.1098/rstb.1887.0011.

[96] Liu B, Liu GH, Hu GP, Sengonca C, Lin NQ, Tang JY, Tang WQ, Lin YZ. 2014. *Bacillus bingmayongensis* sp. nov., isolated from the pit soil of Emperor Qin's Terra-Cotta warriors in China. Antonie Van Leeuwenhoek 105:501–510. doi:10.1007/s10482-013-0102-3. 24370979

[97] Guinebretiere MH, Auger S, Galleron N, Contzen M, De Sarrau B, De Buyser ML, Lamberet G, Fagerlund A, Granum PE, Lereclus D, De Vos P, Nguyen-The C, Sorokin A. 2013. *Bacillus cytotoxicus* sp. nov. is a novel thermotolerant species of the *Bacillus cereus* group occasionally associated with food poisoning. Int J Syst Evol Microbiol 63:31–40. doi:10.1099/ijs.0.030627-0. 22328607

[98] Liu X, Wang L, Han M, Xue Q-h, Zhang G-s, Gao J, Sun X. 2020. *Bacillus fungorum* sp. nov., a bacterium isolated from spent mushroom substrate. Int J Syst Evol Microbiol 70:1457–1462. doi:10.1099/ijsem.0.003673. 32155116

[99] Jung MY, Paek WK, Park IS, Han JR, Sin Y, Paek J, Rhee MS, Kim H, Song HS, Chang YH. 2010. *Bacillus gaemokensis* sp. nov., isolated from foreshore tidal flat sediment from the Yellow Sea. J Microbiol 48:867–871. doi:10.1007/s12275-010-0148-0. 21221948

[100] Laubach CA. 1916. Spore-bearing bacteria in dust. J Bacteriol 1:493–505. doi:10.1128/jb.1.5.493-533.1916. 16558711 PMC378672

[101] Nakamura LK. 1998. *Bacillus pseudomycoides* sp. nov. Int J Syst Bacteriol 48 Pt 3:1031–1035. doi:10.1099/00207713-48-3-1031. 9734060

[102] Milner RJ. 1994. History of *Bacillus thuringiensis*. Agric Ecosyst Environ 49:9–13. doi:10.1016/0167-8809(94)90014-0.

[103] Jimenez G, Urdiain M, Cifuentes A, Lopez-Lopez A, Blanch AR, Tamames J, Kampfer P, Kolsto AB, Ramon D, Martinez JF, Codoner FM, Rossello-Mora R. 2013. Description of *Bacillus toyonensis* sp. nov., a novel species of the *Bacillus cereus* group, and pairwise genome comparisons of the species of the group by means of ANI calculations. Syst Appl Microbiol 36:383–391. doi:10.1016/j.syapm.2013.04.008. 23791203

[104] Miller RA, Beno SM, Kent DJ, Carroll LM, Martin NH, Boor KJ, Kovac J. 2016. *Bacillus wiedmannii* sp. nov., a psychrotolerant and cytotoxic *Bacillus cereus* group species isolated from dairy foods and dairy environments. Int J Syst Evol Microbiol 66:4744–4753. doi:10.1099/ijsem.0.001421. 27520992 PMC5381181

[105] Lechner S, Mayr R, Francis KP, Pruss BM, Kaplan T, Wiessner-Gunkel E, Stewart GS, Scherer S. 1998. *Bacillus weihenstephanensis* sp. nov. is a new psychrotolerant species of the *Bacillus cereus* group. Int J Syst Bacteriol 48:1373–1382. doi:10.1099/00207713-48-4-1373. 9828439

[106] Winter DJ. 2017. rentrez: an R package for the NCBI eUtils API. R J 9:520–526. doi:10.32614/RJ-2017-058.

[107] Barrett T, Clark K, Gevorgyan R, Gorelenkov V, Gribov E, Karsch-Mizrachi I, Kimelman M, Pruitt KD, Resenchuk S, Tatusova T, Yaschenko E, Ostell J. 2012. BioProject and BioSample databases at NCBI: facilitating capture and organization of metadata. Nucleic Acids Res 40:D57–D63. doi:10.1093/nar/gkr1163. 22139929 PMC3245069

[108] Bonis M, Felten A, Pairaud S, Dijoux A, Maladen V, Mallet L, Radomski N, Duboisset A, Arar C, Sarda X, Vial G, Mistou MY, Firmesse O, Hennekinne JA, Herbin S. 2021. Comparative phenotypic, genotypic and genomic analyses of *Bacillus thuringiensis* associated with foodborne outbreaks in France. PLoS One 16:e0246885. doi:10.1371/journal.pone.0246885. 33607651 PMC7895547

[109] Leinonen R, Sugawara H, Shumway M, International Nucleotide Sequence Database Collaboration. 2011. The sequence read archive. Nucleic Acids Res 39:D19–D21. doi:10.1093/nar/gkq1019. 21062823 PMC3013647

[110] Kodama Y, Shumway M, Leinonen R, International Nucleotide Sequence Database Collaboration. 2012. The Sequence Read Archive: explosive growth of sequencing data. Nucleic Acids Res 40:D54–D56. doi:10.1093/nar/gkr854. 22009675 PMC3245110

[111] Tonkin-Hill G, MacAlasdair N, Ruis C, Weimann A, Horesh G, Lees JA, Gladstone RA, Lo S, Beaudoin C, Floto RA, Frost SDW, Corander J, Bentley SD, Parkhill J. 2020. Producing polished prokaryotic pangenomes with the Panaroo pipeline. Genome Biol 21:180. doi:10.1186/s13059-020-02090-4. 32698896 PMC7376924

[112] Page AJ, Taylor B, Delaney AJ, Soares J, Seemann T, Keane JA, Harris SR. 2016. SNP-sites: rapid efficient extraction of SNPs from multi-FASTA alignments. Microb Genom 2:e000056. doi:10.1099/mgen.0.000056. 28348851 PMC5320690

[113] Tavaré S. 1986. Some probabilistic and statistical problems in the analysis of DNA sequences. Lectures on Mathematics in the Life Sciences 17:57–86.

[114] Croucher NJ, Page AJ, Connor TR, Delaney AJ, Keane JA, Bentley SD, Parkhill J, Harris SR. 2015. Rapid phylogenetic analysis of large samples of recombinant bacterial whole genome sequences using Gubbins. Nucleic Acids Res 43:e15. doi:10.1093/nar/gku1196. 25414349 PMC4330336

[115] Paradis E, Claude J, Strimmer K. 2004. APE: analyses of phylogenetics and evolution in R language. Bioinformatics 20:289–290. doi:10.1093/bioinformatics/btg412. 14734327

[116] Paradis E, Schliep K. 2019. ape 5.0: an environment for modern phylogenetics and evolutionary analyses in R. Bioinformatics 35:526–528. doi:10.1093/bioinformatics/bty633. 30016406

